# Large-Scale Computational Discovery of Binding Motifs in tRNA Fragments

**DOI:** 10.3389/fmolb.2021.647449

**Published:** 2021-06-22

**Authors:** Lingyu Guan, Vincent Lam, Andrey Grigoriev

**Affiliations:** ^1^ Department of Biology, Center for Computational and Integrative Biology, Rutgers University, Camden, NJ, United States; ^2^ Department of Molecular Biology and Genetics, Cornell University, Ithaca, NY, United States

**Keywords:** tRNA, tRNA fragments, Argonaute, binding motif, target genes

## Abstract

Accumulating evidence has suggested that tRNA-derived fragments (tRFs) could be loaded to Argonaute proteins and function as regulatory small RNAs. However, their mode of action remains largely unknown, and investigations of their binding mechanisms have been limited, revealing little more than microRNA-like seed regions in a handful of tRFs and a few targets. Here, we identified such regions of potential interaction on a larger scale, using *in vivo* formed hybrids of guides and targets in crosslinked chimeric reads in two orientations. We considered “forward pairs” (with guides located on the 5′ ends and targets on the 3′ ends of hybrids) and “reverse pairs” (opposite orientation) and compared them as independent sets of biological constructs. We observed intriguing differences between the two chimera orientations, including the paucity of tRNA halves and abundance of polyT-containing targets in forward pairs. We found a total of 197 quality-ranked motifs supported by ∼120,000 tRF–mRNA chimeras, with 103 interacting motifs common in forward and reverse pairs. By analyzing T→C conversions in human and mouse PAR-CLIP datasets, we detected Argonaute crosslinking sites in tRFs, conserved across species. We proposed a novel model connecting the formation of asymmetric pairs in two sets to the potential binding mechanisms of tRFs, involving the identified interaction motifs and crosslinking sites to Argonaute proteins. Our results suggest the way forward for further experimental elucidation of tRF-binding mechanisms.

## Introduction

Transfer RNA–derived fragments (tRFs) are an emerging regulatory class of small RNAs. Often detected loaded to Argonaute (Ago) proteins (Kumar et al., 2014; Karaiskos et al., 2015) in RNA-induced silencing complexes, tRFs may function similarly to microRNAs (miRNAs) and control the expression of target genes (Cole et al., 2009; Haussecker et al., 2010; Miyoshi et al., 2010; Burroughs et al., 2011; Li et al., 2012; Maute et al., 2013; Kuscu et al., 2018). The mechanisms of such control remain largely unclear, although recent studies (Kuscu et al., 2018) have identified miRNA-like seed regions, possibly responsible for target binding in several tRFs.

A large-scale screen of such binding has been performed using CLASH (crosslinking, ligation, and sequencing of hybrids), generating sequences of the RNA–RNA hybrid reads formed *in vivo* between small RNA guides and their potential targets in HEK293 cells (Helwak et al., 2013). Originally focused on miRNAs, this dataset has been used to find tRFs in chimeric sequences, with a handful of examples of pairing tRFs with targets (Kumar et al., 2014) with guides located on the 5′ ends and targets on the 3′ ends of hybrids. We refer to chimeras arranged in this orientation as “forward pairs” (Supplementary Figure S1). Previously, we have analyzed a higher stringency subset of forward pairs and predicted 26 binding domains (somewhat equivalent to seed regions) of tRFs (Guan et al., 2020), including several cases that were validated experimentally.

Given the value of CLASH in studying putative tRF-binding mechanisms, we undertook a more comprehensive analysis of this dataset in the current study. We considered “reverse pairs” (tRFs on the 3′ ends of the reads and targets on the 5′ ends) and compared their tRF–target pairs to those identified in forward pairs (Supplementary Figure S1). Reverse miRNA–mRNA pairs that are comparable in abundance to forward pairs have been reported in CLASH (Helwak et al., 2013). Our analyses expanded this observation, revealing unexpected differences in sequence composition, parent tRNAs, and other characteristics that distinguished the two binding orientations. We analyzed them in the context of the Ago structure and binding to guides and targets, using PAR-CLIP data in human and mouse cells (Hafner et al., 2010; Sarshad et al., 2018). Our results suggest a model connecting the formation of forward and reverse chimeras with guide–target interactions. We provide the details of 197 quality-ranked motifs supported by ∼120,000 tRF–mRNA interactions, paving the way for further experimental elucidation of tRF-binding mechanisms.

## Methods

### Analysis of Crosslinking, Ligation, and Sequencing of Hybrids Data

We downloaded CLASH data (Helwak et al., 2013) from the SRA database (SRR959751 to SRR959759). We followed the analysis pipeline in our earlier study (Guan et al., 2020) but this time included tRFs on the 3′ end of a CLASH read by looking for the longest tRNA-matching sequence ending at the last nucleotide of the read (Supplementary Figure S1). We downloaded the secondary structure information for reference tRNAs from tRNAdb (Juhling et al., 2009) and GtRNAdb (Chan and Lowe, 2009).

We detected motifs as described previously (Guan et al., 2020), excluding targets with runs ≥5 continuous Ts. We relaxed the requirement of two supporting reads per tRF–target pair, aiming to detect all possible motifs common between the two orientations and combine weak evidence in the two orientations to generate motifs.

### Analysis of PAR-CLIP Data

We downloaded high-throughput sequencing datasets for Ago1 to Ago4 PAR-CLIP in HEK293 cells (SRR048973 to SRR048979) (Hafner et al., 2010) and for Ago2 PAR-CLIP in mouse ESCs and C2C12 myocytes (Sarshad et al., 2018) from the SRA database. We used the FASTX-Toolkit (http://hannonlab.cshl.edu/fastx_toolkit/) to remove adapters and Bowtie 1.0.1 (Langmead et al., 2009) to align the reads to tRNA references in the end-to-end mode, allowing one T-to-C mismatch and giving preference to perfect matches, as in the earlier tRF analysis (Kumar et al., 2014). tRFs shorter than 16 nt were excluded. We detected mismatches between PAR-CLIP reads and tRNA sequences, normalized to reads per million in each Ago-PAR-CLIP sample, and combined the results of different samples to determine T→C conversion site frequencies in tRFs. Mouse tRFs fully matching human tRNAs were used to map T→C sites from mouse tRFs to human tRFs. These analyses were repeated for miRNAs to compare the results.

### Analysis of icSHAPE Data

icSHAPE reactivity scores for cytoplasmic RNAs in HEK293 cells were downloaded from the UCSC genome browser (Sun et al., 2019). We collected the scores for each nucleotide position in the target sequence identified in CLASH chimeric reads and in the flanking sequence. Target sequences without valid icSHAPE scores (such as introns) were excluded from the analysis.

## Results

### Identification of Forward and Reverse tRF–Target Pairs

Overall, we identified ∼4.7 million chimeric CLASH reads containing tRFs on the 5′ end and ∼3.5 million reads containing tRFs on the 3′ end (Table 1), excluding reads having ≥80% of their length mapped to a tRNA. We first considered tRFs pairing with other ncRNAs. Most of the pairs were formed between tRFs and rRNA, with some strong biases (Table 1, see also our tRF notation there). For example, reads with tRF-3p or tRF-3t showed some sevenfold excess in forward vs. reverse pairs with rRNA. For miRNA and other ncRNAs paired with tRFs, the difference in the numbers of reads containing forward and reverse pairs was more modest, up to 1.5-fold. We also found chimeras with tRFs on both ends of the reads (Supplementary Table S1). A total of 120,805 cases of pairs of tRFs from different tRNA genes pointed to potential intermolecular interactions of two tRNAs or their tRFs. However, we also observed 493,957 occurrences of disjoint fragments from the same tRNA, possibly formed due to intramolecular interactions at the stems in the same tRNA molecule being fragmented, or stem-like interactions of two independently produced tRFs. However, all such tRF–tRF pairs may also be artifacts of chimera formation or sequencing.

**TABLE 1:** Numbers of reads supporting hybrids with specific types of targets in forward and reverse pairs of tRFs.

	Guides^a^	All aligned reads^b^ (<80% match with tRNA)	mRNA^c^	rRNA	miRNA	Other^d^ ncRNA
Forward pairs	tRF-5p	37,459/662,433	12,282 (5,754)	20,065 (15,535)	2,606 (2,297)	2,506 (1,075)
	tRF-5i	25,531/57,921	5,190 (2,840)	19,556 (16,654)	349 (295)	436 (184)
	tRF-3p	909,963/1,599,089	75,163 (54,562)	809,273 (794,288)	6,261 (5,701)	19,266 (16,271)
	tRF-3i	4,754/45,863	1,095 (671)	2,859 (2,250)	595 (547)	205 (109)
	tRF-3t	131,129/281,072	10,425 (7,550)	115,997 (112,457)	1,903 (1,686)	2,804 (1,981)
	tRF-i	89,822/2,048,367	16,314 (10,100)	65,365 (53,181)	4,515 (3,841)	3,628 (2,237)
Reverse pairs	tRF-5p	46,059/210,383	10,046 (7,531)	29,460 (26,318)	4,425 (4,092)	2,128 (1,823)
	tRF-5i	53,354/188,008	9,747 (7,874)	40,780 (39,000)	1,890 (1,785)	937 (788)
	tRF-3p	170,215/1,667,846	48,859 (39,734)	101,831 (92,839)	4,171 (3,740)	15,354 (13,368)
	tRF-3i	15,090/47,882	895 (660)	13,877 (13,110)	195 (168)	123 (80)
	tRF-3t	23,720/222,031	5,404 (4,157)	16,145 (13,701)	930 (784)	1,241 (957)
	tRF-i	123,266/1,194,325	11,586 (8,115)	104,395 (94,232)	4,167 (3,569)	3,118 (2,312)

a Notation for tRF types.

tRF-5p start in the first 5 nt of tRNA genes and end before the anticodon loop.

tRF-5i start in the first 5 nt of tRNA genes and end in the anticodon loop.

tRF-3p end in the last 5 nt of tRNA genes with CCA additions and start after the anticodon loop.

tRF-3i end in the last 5 nt of tRNA genes with CCA additions and start in the anticodon loop.

tRF-3t end at least 3 nt downstream of tRNA genes and also include fragments typically called tRF-1 (starting after the end of tRNA genes).

tRF-i do not fit into the above categories and start and end in the internal regions of tRNA genes.

In general, a notation for each tRF is provided as ***host_gene-[X] -type-start-end***, where X stands for the tRNA gene origin: [N] for nuclear, [M] for mitochondrial, or [NM] for cases when nuclear and mitochondrial cannot be distinguished. Start and end correspond to the coordinates on the tRNA gene, including introns, if present in tRNA. CCA is assumed added to tRF-3p, while 3′ trailer is added to tRF-3t.

b Numbers before slash indicate the interactions of tRFs and annotated targets (mRNAs, rRNAs, miRNAs, and other ncRNAs). Numbers after slash indicate all CLASH reads containing tRFs.

c Numbers in parentheses correspond to the hybrids supported by at least two reads.

d Excluding tRNA.

The tRF–ncRNA pairs and the observed biases may warrant further study. We detected tRFs exactly matching tRNA introns, although only one, from TyrGTA-001, had >100 reads, all paired with rRNA. However, as our main focus was on the pairs of tRFs and mRNAs, we excluded the above chimeras from further analysis.

Different tRF types are formed from the same tRNAs, but forward chimeras reveal a paucity of 5′ tRNA halves.

**FIGURE 1:**
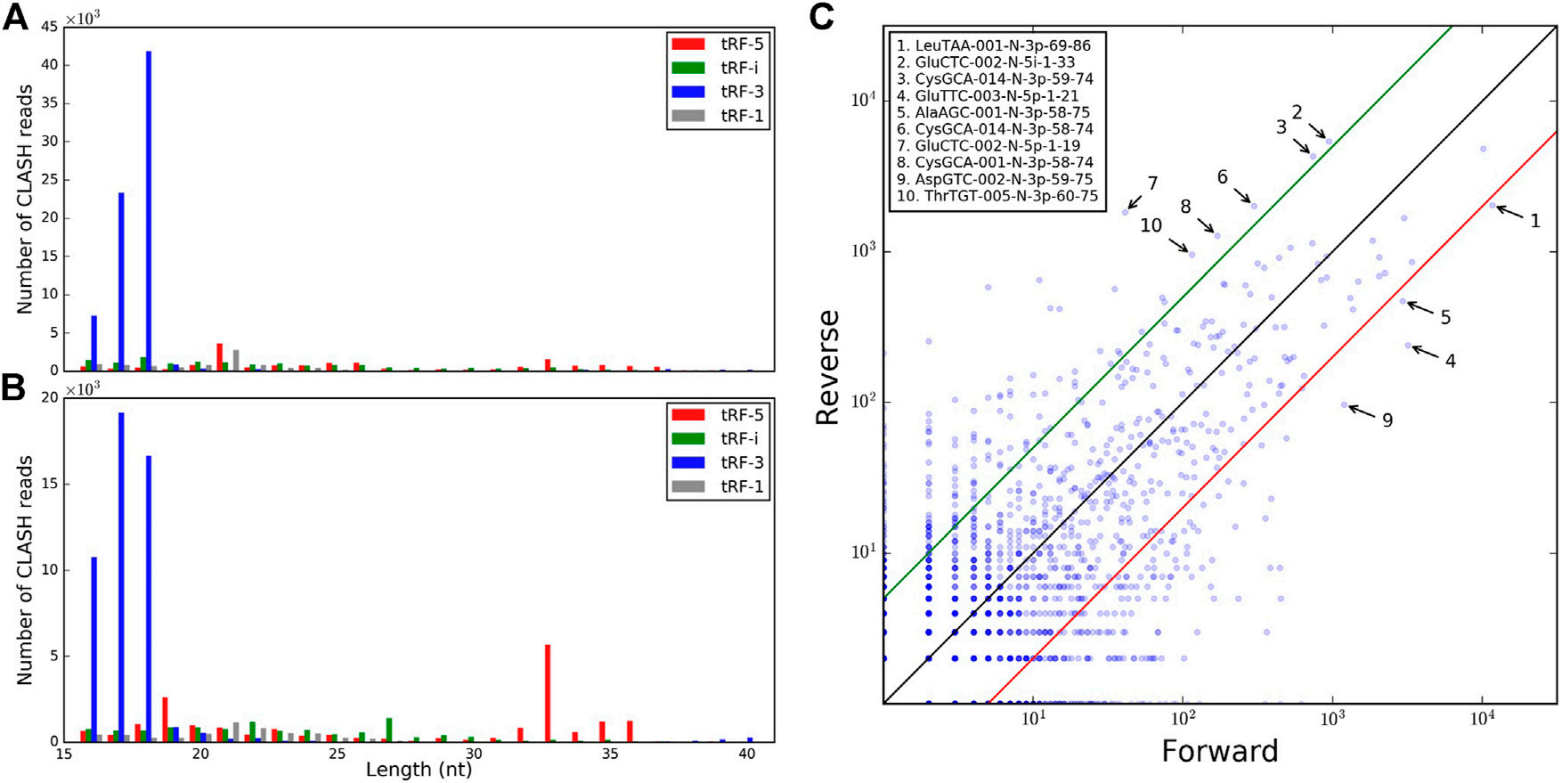
Comparison of the tRFs identified in forward and reverse pairs. The tRF length distributions are shown for different types of tRFs identified in **(A)** forward pairs and **(B)** reverse pairs. **(C)** Relative frequencies of tRFs common to forward and reverse chimeras. The 10 most abundant tRFs with the highest differential frequencies in the two orientations (fold change >5) are labeled.

We next considered tRFs based on their origin within a tRNA gene. Although tRF-3s were the most abundant with 18 nt being the mode of the length distribution overall, such reads were depleted by >50% in reverse pairs and 17 nt tRF-3s became slightly more prevalent (Figures 1A,B). A negligible amount of longer tRF-3s (39–40 nt, <1%) was seen in the reverse set. The other three types of tRFs were less abundant than tRF-3s in the two sets. Nevertheless, in the reverse orientation, we observed some abundant long tRF-5s (33–36 nts, Figure 1B and Table 1). These tRF-5s, with 3′ border primarily cut in the anticodon loops, are believed to be processed by endonuclease angiogenin under stress conditions (Fu et al., 2009; Yamasaki et al., 2009). They are often named “tRNA halves,” or “5′ tiRNAs.” Here, for a consistent short notation, we call them “tRF-5i” to differentiate tRNA halves from shorter tRF-5p, which had their 3′ borders cleaved before the anticodon loops. One example (GluCTC-002-N-5i-1-33, see Table 1 for full notation) is shown in Figure 1C, having a 10-fold lower abundance in forward pairs. Different from other tRFs, tRF-5i would be expected to contain a 2′,3′-cyclic phosphate on their 3′ end if they are produced by angiogenin (Shigematsu and Kirino, 2020). The CLASH procedure [steps 34–37 (Helwak et al., 2013)] potentially preserving some cyclic phosphate 3′ ends within Ago might lead to a less efficient ligation to their targets and be the reason why tRF-5i were less frequent in the forward set (∼2-fold across different target groups; 2.5-fold with two-read support, Table 1).

We assessed whether tRFs from different tRNAs were found at similar frequencies on the 5′ and 3′ ends of the chimeric reads. Many isoforms, with slight variations in tRF length, akin to isomiRs (Morin et al., 2008), showed different abundance in the two sets, by 5- to 10-fold (including CysGCA, GluTTC, and GluCTC, Figure 1C). Combining all isoforms of all isodecoders, the difference between forward and reverse chimeras was less drastic (Supplementary Figure S2A, high Pearson R = 0.85, *p*-value = 7.25E-64) and showed different outliers (Supplementary Figure S2B). Hence, binding tRFs at the level of isodecoders may not reflect the full complexity of the individual tRF isoform distribution.

### tRF Targets in Forward Pairs Have a Unique polyT Group

**FIGURE 2:**
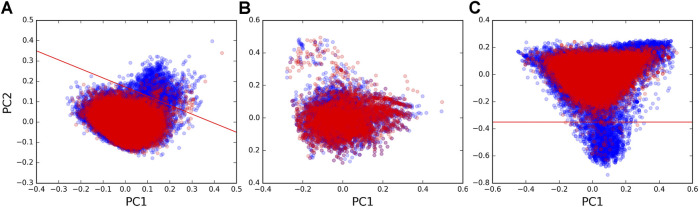
PCA plots of dinucleotide composition. Plots of the first vs. second principal components calculated from dinucleotide frequencies (counting all overlapping dinucleotides) are shown for **(A)** tRF–mRNA chimeric sequences, **(B)** tRF sequences, and **(C)** mRNA target sequences that were identified in forward pairs (blue points) and reverse pairs (red points). Red lines separate the region enriched with polyT runs from the rest of the sequences.

We next sought to uncover possible differences in the sequences of the guides and the targets of forward and reverse chimeras. We performed principal component analysis (PCA, using *scikit-learn* available at https://scikit-learn.org/) to assess possible sequence differences using the dinucleotide composition of chimeras as a metric. We found that the majority of pairs in the forward and in the reverse set occupied the same regions on the PCA plot, suggesting that chimeras in both directions captured tRF–target interactions of similar sequence composition. However, a region was seen on the plot with some pairs that almost exclusively originated from the forward set (Figure 2A). We extracted 1,927 tRF–target pairs from this region (above the red line, Figure 2A) and found that target sequences in those pairs contained polyT runs (in fact, 46.77% of nucleotides in these target sequences were Ts vs. 21.22% in other targets). Such differences could also be seen when plotting the dinucleotide composition of only target sequences (Figure 2C), while tRF sequences had much smaller differences between the forward and reverse sets (Figure 2B).

In order to see if polyT was a distinguishing characteristic of targets in forward pairs, we examined where chimeras with polyTs were located on the PCA plot for target sequences in all forward and reverse tRF–target pairs. Of the 92,123 unique tRF–target pairs, 1,581 had a 5 nt polyT as the longest run of Ts in their target sequences. A total of 2,719 had at least one polyT >5 nts in length. The tRF–target pairs containing longer polyT runs in the last 10 nts of target sequences (Supplementary Figure S3) were frequently in the region populated by forward pairs (below the red line in Figure 2C). On the contrary, the points in Supplementary Figure S3 with shorter polyT runs located away from the 3′ end of targets were distributed across all regions of the PCA plot. The two-read support of polyT chimeras (28%) was lower than that of overall mRNA (Table 1), and their paucity among miRNA targets (145 pairs) suggested polyTs were a tRF-CLASH–specific artifact.

### Chimeras in Both Orientations Contain Similar tRF Motifs

For every tRF, we followed our earlier approach (Guan et al., 2020) to identify motifs separately in both chimera orientations. We observed polyT-containing sequences to introduce bias and mask the otherwise significant motifs and excluded them from motif search in the targets of a given tRF. Although forward and reverse motifs varied by a few nucleotides in length, their overall sequence composition was quite similar (Supplementary Figure S4). We considered forward and reverse chimeras to be two independent sets of biological constructs, with their own biases, and found 103 common motifs in both (including ten identical motifs in the two sets, 91 overlapping ≥4 nt, and two motifs by only a couple of nt). This (i) further supported the validity of their detection in our computational pipeline and (ii) strongly indicated that motif sequences were likely responsible for binding tRFs to targets. Given such agreement and that some tRFs had insufficient numbers of different binding targets in one orientation, we combined forward and reverse reads and identified a total of 197 significant motifs (Supplementary Figure S5). These comprised 150 forward and 132 reverse motifs. For three tRFs forward and reverse motifs did not overlap.

Motifs were not found in all cases. 7.53% tRFs did not have sufficient numbers of different CLASH targets to infer binding motifs at the chosen threshold of significance (E-value < 0.01) determined by MEME (Bailey et al., 2009). For 4.93% tRFs, their target motifs did not match back with tRFs by FIMO (Grant et al., 2011) at *p*-value < 0.001. Also, while some target sequences were ligated to tRFs in CLASH, their binding modes remained unclear due to a lack of matches with the tRF motifs (60,414 out of 207,006 tRF–mRNA chimeras).

### tRF Motifs Are Compatible With Ago Structure

4-Thiouridine–modified residues crosslinking with RNA-binding proteins often change to cytidines in PAR-CLIP. A previous analysis of human PAR-CLIP has found the primary Ago-crosslinking sites (T→C conversions) at positions 8–12 of tRFs (Kumar et al., 2014). The conclusion on the Ago-crosslinking sites being depleted in (and adjacent to) target binding regions has been reached in that work based on the hypothesis that tRFs used miRNA-like seeds (2–8 nucleotides on the 5′ end) to interact with targets and only considered forward pairs.

**FIGURE 3:**
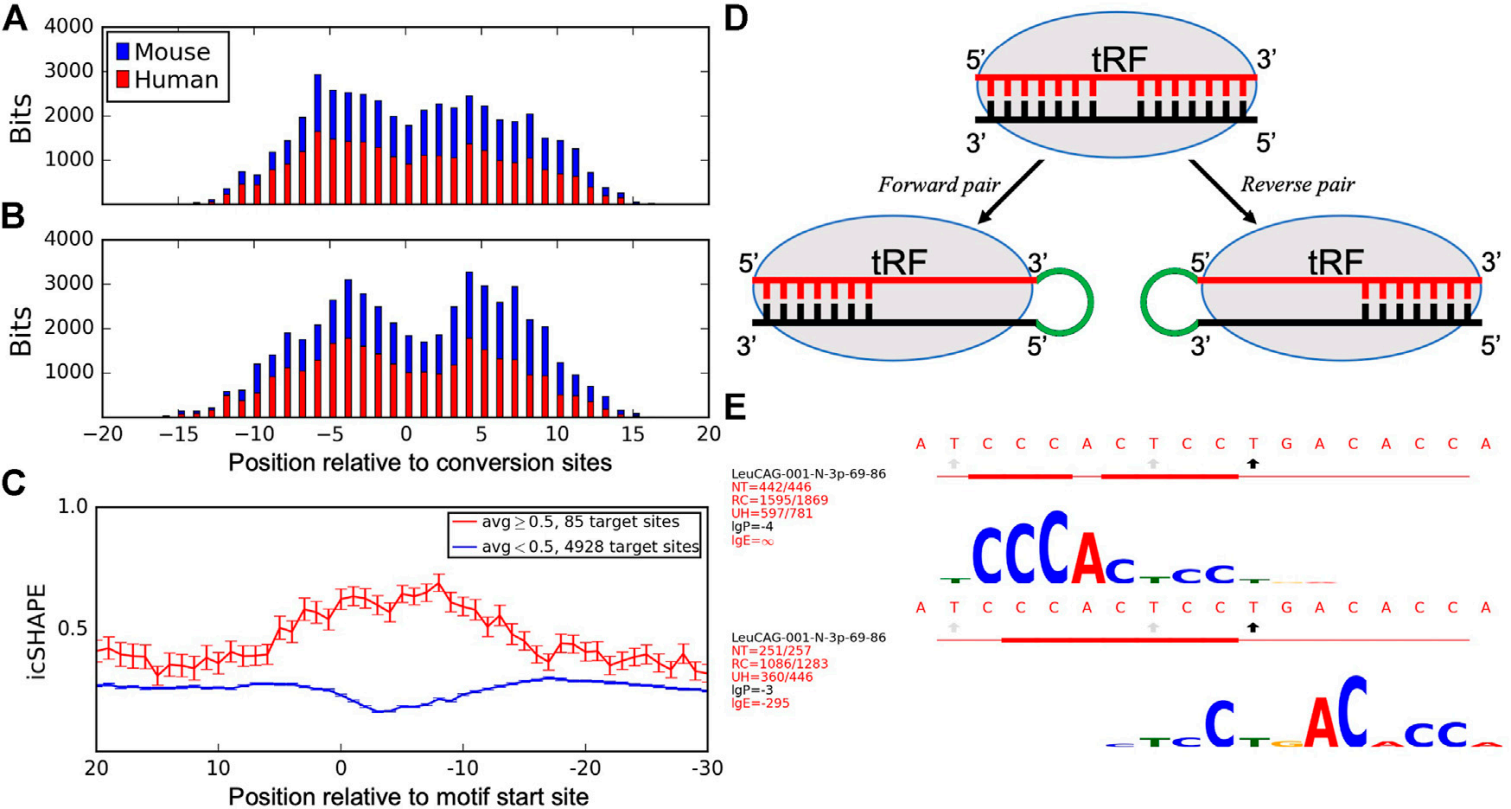
Analyses of Ago-crosslinking and target binding sites. Histograms show the cumulative bitscores of motifs in **(A)** forward and **(B)** reverse pairs relative to the most frequent T→C conversion sites identified for all motif-containing tRFs in human cells (blue) and identical tRFs in mouse cells (red). Bitscores (heights of logo symbols, reflecting the complementary match frequencies between tRFs and target nucleotides) for every position in the motif were added up in respective positions of all motif-containing tRFs relative to their conversion sites as a measure of target binding frequencies in these positions. tRFs are aligned to the most frequent conversion sites and shown from 5′ to 3′. **(C)** icSHAPE scores of target sites relative to the predicted interacting motifs. Activity scores are averaged for every nucleotide of all unique mRNA targets and separated into single-stranded (red) and double-stranded (blue) regions formed at the motif site. mRNA targets are aligned to the start of motif-binding sites and shown from 3′ to 5'. **(D)** A model describing how Ago-crosslinking and target binding results may be connected to the observed asymmetries of tRF and target pairs and their motifs. **(E)** Motifs found for CysGCA tRF in the forward **(top)** and reverse **(bottom)** chimeras, see the legend of Supplementary Figure S5 for details.

Here, we expanded the analysis to include available Ago PAR-CLIP datasets in human (Hafner et al., 2010) and mouse (Sarshad et al., 2018) cells. We compared the species-specific T→C conversion patterns to the identified motifs to determine the association between target binding sites and Ago-crosslinking sites. We aligned all tRFs placing the most frequent T→C conversion site at position 0 and showed the cumulative information content of the motifs. The peaks of cumulative bitscores, corresponding to the most conserved motif positions (tallest logo letters in Supplementary Figure S5, indicating frequent binding with different targets), were 5–6 nts away on both sides from the most frequent conversion site, which showed a drop in binding (Figures 3A,B). We then plotted these motif bitscores relative to top conversion sites in the same tRFs in mouse Ago2 PAR-CLIP data and observed the same pattern as in humans. We separated forward and reverse pairs in these plots and saw that tRF motifs in forward pairs showed higher bitscores upstream and in reverse pairs downstream of the T→C conversion site in both species. This interesting difference is consistent with the Ago structure and may explain the formation of forward and reverse pairs (see Discussion).

### Characterization of tRF Protein–Coding Targets

We observed various mRNAs targeted by tRFs in high numbers (Supplementary Table S2). In both orientations, CDS and 3′UTR were the most frequent targeted regions, followed by 5′UTR and intronic regions [we have discussed the implication of intronic targets of tRFs previously (Guan et al., 2020)].

The most abundant interactions were formed between CysGCA tRF-3p (including tRFs mapped to three different CysGCA tRNA isodecoders) and HIST2H2AA3, which accounted for 17% of all reverse pairs. The proportion of these interactions dropped to 2.7% in forward pairs, but they were still among the top five most abundant pairs. >97% of the interactions of HIST2H2AA3 were in its 3′UTR.

Ribosomal proteins (RPs) were frequent targets of tRFs in both directions (Supplementary Table S2). RPL35A, the most abundant RP as noted earlier (Guan et al., 2020), was targeted in both orientations by two tRFs derived from two ThrAGT tRNAs, with ThrAGT-005 dominating in the forward direction and ThrAGT-006 (1 nt difference from 005) in the reverse direction. Another abundant RP—RPL4—was most frequently targeted by IleTAT tRF-3t in forward pairs, whereas in reverse, GlyGCC tRF-5p became its top guide.

Previous studies have revealed different RPs as targets of tRFs in humans (Kim et al., 2017) and fruit flies (Luo et al., 2018). A 22 nt LeuCAG tRF-3p has been shown to upregulate the expression of RPS28 by binding to and unfolding the secondary structure of the mRNA. However, it was posited to act without the involvement of Ago, as this isoform was not associated with Ago in northern blot (Kim et al., 2017). Consistent with this observation, we only detected a handful of chimeric reads containing the 22 nt LeuCAG isoform. Instead, the major isoform of LeuCAG tRF-3p in CLASH was 18 nt, present in 2,136 reads.

We analyzed intramolecular secondary structures of tRF targets to investigate if Ago1-loaded tRFs may act similar to LeuCAG. We utilized the icSHAPE (*in vivo* click-selective 2′-hydroxyl acylation and profiling experiments) dataset for the cytoplasm of HEK293 cells (Sun et al., 2019). Having aligned all target genes so that their motif starts were at the same position (zero on the *x*-axis), we plotted the average icSHAPE score for each target position (Figure 3C). Genes were separated into two groups: with motif in double-stranded (DS, average icSHAPE score <0.5 in the motif region, blue line in Figure 3C) and single-stranded (SS, score ≥0.5, red line) regions. Both lines clearly separated at the motif site, but not in the flanking regions. The changes in icSHAPE scores relative to the target flanking regions were significant compared to a random set of 12-mers from target genes in the DS (*p*-value = 1.58E-14) and SS (*p*-value = 1.36E-5) sites. This suggested that the structure (SS or DS) of tRF–target binding regions was much better defined than the surrounding areas in targets. Such structures showed a clear bias as 98.3% of target sites with available icSHAPE data (4928/5013) were in DS regions with the largest separation right over the motif (*p*-value < E-16 from position 0 to 10, z-test).

## Discussion

Transfer RNA–derived fragments have recently attracted significant attention as a new class of regulatory small RNAs. However, experimental studies have mostly focused on only a few tRFs and computationally predicted targets. The CLASH method has provided data to study guide–target pairs formed *in vivo*. Our previous proof-of-principle analysis of targeting mechanisms using the CLASH dataset has identified tRF binding regions in 26 tRFs (Guan et al., 2020). Here, we utilized this dataset to conduct a more comprehensive analysis, including tRFs and targets ligated in CLASH chimeras in both forward and reverse orientations.

We identified millions of reads containing fragments that could be perfectly mapped to tRNAs. tRF-i, which indicate tRFs not covering the 5′ or 3′ end of tRNAs, were the most abundant type of tRF found in the CLASH reads. However, these tRF-i did not show efficient binding to targets, <1% of tRF-i paired with mRNA/ncRNA, and these interactions were much weaker than those of other types of tRFs, as shown earlier (Guan et al., 2020). We detected tRFs produced from tRNA introns, which have not been reported previously. However, with only one tRF >100 reads and no motifs, their significance is unclear. The most abundant types of tRFs ligated to targets in chimeras were tRF-3p of length 18 nt and 17 nt (Figures 1A,B) in forward and reverse sets, respectively, different from earlier reports (Kumar et al., 2014; Guan et al., 2020).

We observed many similarities but also noticeable differences between the two orientations of tRF–target chimeras. Longer tRFs of 33–36 nt were overrepresented in the reverse set (Figure 1B). Such tRF-5i include the 5′ tRNA end, with the 3′ end suggested to be cleaved in the anticodon loop by angiogenin (Fu et al., 2009; Yamasaki et al., 2009). We observed the presence of polyTs in the targets of forward pairs. These polyT sequences were often seen at the 3′ ends of CLASH reads, and they could be mapped to T-rich regions in certain transcripts. However, it is unclear whether they reflect a genuine feature of a specific group of tRF targets, only seen in the forward pairs, or represent artifacts caused by the experimental library preparation or sequencing errors (their interference with motif searches suggests the latter). In any case, we observed cases of a clear asymmetry of the paired tRFs and their targets in both orientations.

Such asymmetry is likely related to (yet unknown) constraints resulting from the structural properties of Ago and its binding to guides and targets. A recent structure of Ago2 interacting with miRNA (Fabian, 2019; Sheu-Gruttadauria et al., 2019) has indicated a binding gap in the miRNA positions 9–12 (past the seed region), followed by further target binding in a “supplementary chamber” of Ago2. The primary Ago-crosslinking sites reported at positions 8–12 of tRFs (Kumar et al., 2014) correspond to this gap (Figures 3A,B). We propose a possible model (Figure 3D) that may account for some of the asymmetry, connecting it with other observations. A higher motif bitscore (*p*-value = 5.23E-8, binomial test) upstream of the conversion site (Figure 3A) indicates a higher frequency of complementary matches to different targets bound to these motif nucleotides. Such biased binding might result in an increased availability (exacerbated by the CLASH procedure) of the 3′ end of such tRFs for ligation, compared to the target-bound 5′ end, resulting in a higher frequency of forward chimeras. The opposite arrangement in the reverse pairs would correspond to the targets more frequently bound at the 3′ end of a tRF (Figure 3B, *p*-value = 7.09E-5, binomial test), downstream of the conversion site, and ligation events upstream of such a tRF. The latter outcome seems less frequent for mRNA targets (Table 1), except for tRF-5i, whose forward ligation may be hindered by the 2′,3′-cyclic phosphate on their 3′end if they are produced by angiogenin (Shigematsu and Kirino, 2020).

Thus, some tRFs may interact with different targets using different parts of an extended binding region (Figure 3E). Consistent with this model are cases of such overlapping motifs for forward and reverse orientations in the same tRFs in Supplementary Figure S5: 60 forward motifs start upstream of reverse motifs vs. 25 cases of reverse motifs starting upstream (*p*-value = 1.87E-4, binomial test, we call this “upstream–downstream test” below). Will the observed divergence in the binding region lead to different effects on the targets? Experimental validation of such extended binders should shed further light on the tRF interaction mechanisms. This may also be relevant to miRNAs, as a large number of them found in the original CLASH report with extended binding regions (Helwak et al., 2013) have been shown (Agarwal et al., 2015) to produce weaker downregulation effects on mRNA, compared to miRNAs with canonical seed regions. Supporting this, the upstream–downstream test showed even higher bias in miRNA (53 vs. 20 cases, *p*-value = 1.42E-4). Then, compatible with Figures 3A,B, forward miRNA–mRNA pairs had frequent binding upstream (*p*-value = 3.59E-5), while reverse pairs were slightly more frequent downstream of the conversion sites (not significant, *p*-value = 0.32) in both human and mouse cells. Such potential for other modes of action is supported by our finding that tRF targets have a well-defined secondary structure compared to their flanking regions and are predominantly double-stranded (Figure 3C). Based on this, we suggest that some of the tRF–target interactions may resemble those that are described for LeuCAG tRF-3p and RPS28 (Kim et al., 2017), binding to and unfolding the secondary structure of target mRNA.

A few motifs contradicted our model, but they were not strongly supported by data, e.g., in LysCTT-003-N-3p (Supplementary Figure S5). The forward motif was close to the 3′ end of the tRF, consistent with our earlier report (Guan et al., 2020), but we also found the reverse motif on the 5′ end, which was much less significant and based only on 39 reads. Additionally, for many tRFs, there were no differences between their interacting target motifs or other properties in forward and reverse sets. The motifs we identified independently using forward or reverse pairs often overlapped and closely resembled each other. Take an example of LeuTAA-001-N-3p-69-86, named tRF-3009a (Kumar et al., 2014), one of the tRFs with experimentally validated seed regions (Kuscu et al., 2018). We obtained the same motif for this tRF in forward and reverse sets, suggesting a binding region at 2–10 nt in the tRF upstream of the Ago crosslinking indicated by high-frequency T→C conversions at position 11 in human PAR-CLIP datasets (Supplementary Figure S5). It is also consistent with how substitutions in the target matching 2–8 seeds in the tRF affected the repression function of the tRF on mutated targets [see Figure 4C in Kuscu et al. (2018)].

Another prediction matched a 21 nt long tRF-3t, recently identified in Ago (known as miR-1983), straddling the 3′ border and including the 3′ trailer of IleTAT tRNA (Hasler et al., 2016). Having previously found its significant forward motif, here we observed the same motif in reverse pairs, finding a complementary motif match in all three experimentally determined targets of IleTAT-005-N-3t (Hasler et al., 2016).

A total of 171 motifs were added in this work to the 26 motifs identified earlier using stringent forward hybrids (Guan et al., 2020), with >50% of the motifs common/overlapping in both orientations. We ranked the motifs by multiple parameters (number of unique interacting target sequences, total read support, *p*-value or E-value of motifs, etc.) and provided lists of abundant tRF targets (Supplementary Table S2). The higher confidence motifs (with red fonts designating entries in the top 1/3 for each parameter in Supplementary Figure S5) suggest a path to direct experimental validation of their role in tRF–target binding. These could also be conveniently queried in a database that we are preparing for public release. During the review of this paper, our work on ribosomal RNA fragments was published (Guan and Grigoriev, 2021). It provided further details on distributions of small RNAs and their targets in CLASH hybrids.

## Data Availability

The original contributions presented in the study are included in the article/Supplementary Material, and further inquiries can be directed to the corresponding author.
