# Supplementary material for: Large-Scale Computational Discovery of Binding Motifs in tRNA Fragments
Source: Front Mol Biosci. 2021 Jun 22;8:647449. doi: 10.3389/fmolb.2021.647449 (PMC8258673; doi:10.3389/fmolb.2021.647449)
Supplement: Supplementary file 1 [file DataSheet2.PDF]

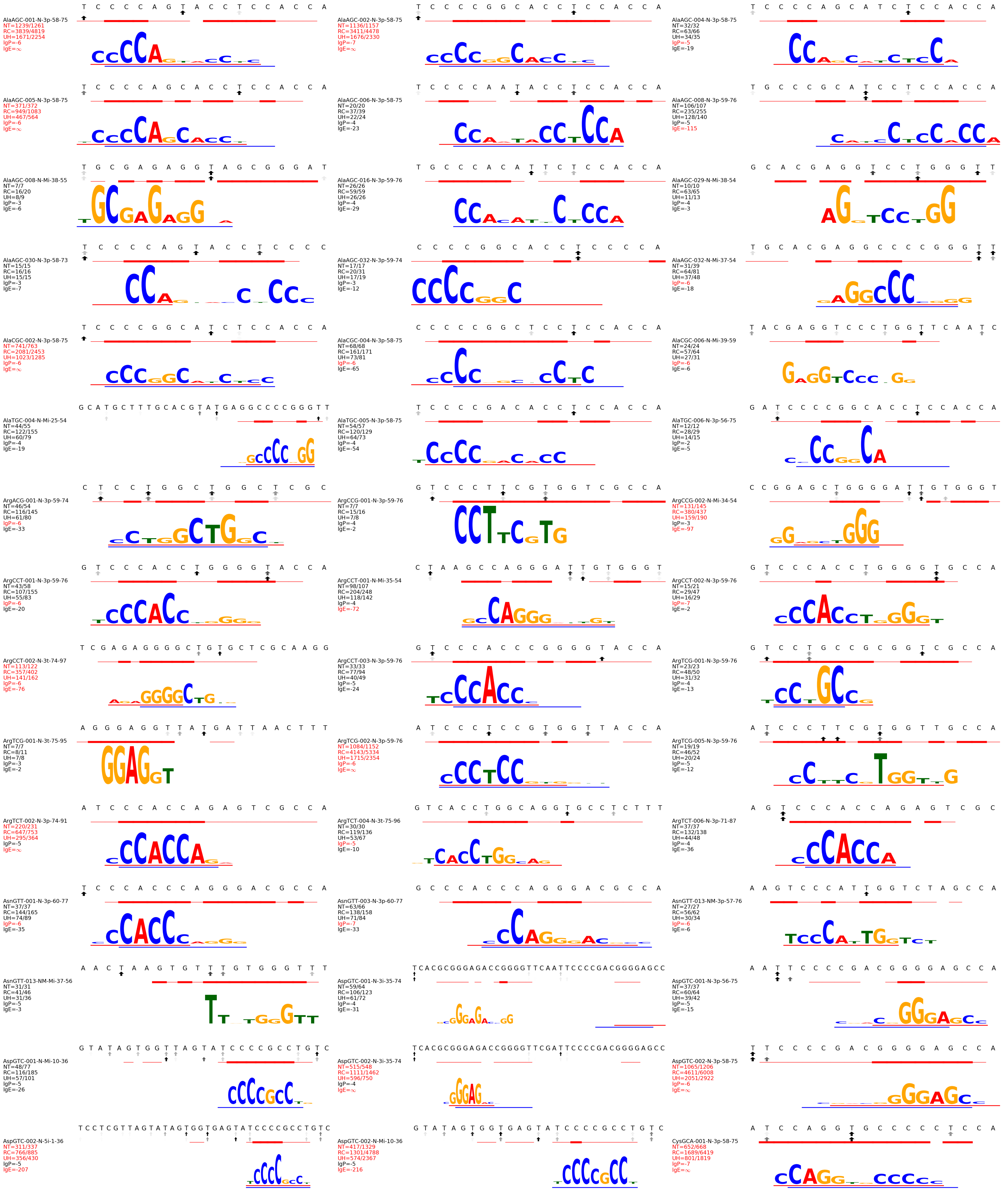

AlaAGC-001-N-3p-58-75 NT=1239/1261 RC=3839/4819 UH=1671/2254 lgP=-6 lgE=∞
AlaAGC-002-N-3p-58-75 NT=1136/1157 RC=3411/4478 UH=1676/2330 lgP=-7 lgE=∞
AlaAGC-004-N-3p-58-75 NT=32/32 RC=63/66 UH=34/35 lgP=-5 lgE=-19
AlaAGC-005-N-3p-58-75 NT=371/372 RC=949/1083 UH=467/564 lgP=-6 lgE=∞
AlaAGC-006-N-3p-58-75 NT=20/20 RC=37/39 UH=22/24 lgP=-4 lgE=-23
AlaAGC-008-N-3p-59-76 NT=106/107 RC=235/255 UH=128/140 lgP=-5 lgE=-115
AlaAGC-008-N-Mi-38-55 NT=7/7 RC=16/20 UH=8/9 lgP=-3 lgE=-6
AlaAGC-016-N-3p-59-76 NT=26/26 RC=59/59 UH=26/26 lgP=-4 lgE=-29
AlaAGC-029-N-Mi-38-54 NT=10/10 RC=63/65 UH=11/13 lgP=-4 lgE=-3
AlaAGC-030-N-3p-58-73 NT=15/15 RC=16/16 UH=15/15 lgP=-3 lgE=-7
AlaAGC-032-N-3p-59-74 NT=17/17 RC=20/31 UH=17/19 lgP=-3 lgE=-12
AlaAGC-032-N-Mi-37-54 NT=31/39 RC=64/81 UH=37/48 lgP=-6 lgE=-18
AlaCGC-002-N-3p-58-75 NT=741/763 RC=2081/2453 UH=1023/1285 lgP=-6 lgE=∞
AlaCGC-004-N-3p-58-75 NT=68/68 RC=161/171 UH=73/81 lgP=-6 lgE=-65
AlaCGC-006-N-Mi-39-59 NT=24/24 RC=57/64 UH=27/31 lgP=-6 lgE=-6
AlaTGC-004-N-Mi-25-54 NT=44/55 RC=122/155 UH=60/79 lgP=-4 lgE=-19
AlaTGC-005-N-3p-58-75 NT=54/57 RC=120/129 UH=64/73 lgP=-4 lgE=-54
AlaTGC-006-N-3p-56-75 NT=12/12 RC=28/29 UH=14/15 lgP=-2 lgE=-5
ArgACG-001-N-3p-59-74 NT=46/54 RC=116/145 UH=61/80 lgP=-6 lgE=-33
ArgCCG-001-N-3p-59-76 NT=7/7 RC=15/16 UH=7/8 lgP=-4 lgE=-2
ArgCCG-002-N-Mi-34-54 NT=131/145 RC=380/437 UH=159/190 lgP=-3 lgE=-97
ArgCCT-001-N-3p-59-76 NT=43/58 RC=107/155 UH=55/83 lgP=-6 lgE=-20
ArgCCT-001-N-Mi-35-54 NT=98/107 RC=204/248 UH=118/142 lgP=-4 lgE=-72
ArgCCT-002-N-3p-59-76 NT=15/21 RC=29/47 UH=16/29 lgP=-7 lgE=-2
ArgCCT-002-N-3t-74-97 NT=113/122 RC=357/402 UH=141/162 lgP=-6 lgE=-76
ArgCCT-003-N-3p-59-76 NT=33/33 RC=77/94 UH=40/49 lgP=-5 lgE=-24
ArgTCG-001-N-3p-59-76 NT=23/23 RC=48/50 UH=31/32 lgP=-4 lgE=-13
ArgTCG-001-N-3t-75-95 NT=7/7 RC=8/11 UH=7/8 lgP=-3 lgE=-2
ArgTCG-002-N-3p-59-76 NT=1084/1152 RC=4143/5334 UH=1715/2354 lgP=-6 lgE=∞
ArgTCG-005-N-3p-59-76 NT=19/19 RC=46/52 UH=20/24 lgP=-5 lgE=-12
ArgTCT-002-N-3p-74-91 NT=220/231 RC=647/753 UH=295/364 lgP=-5 lgE=∞
ArgTCT-004-N-3t-75-96 NT=30/30 RC=119/136 UH=53/67 lgP=-5 lgE=-10
ArgTCT-006-N-3p-71-87 NT=37/37 RC=132/138 UH=44/48 lgP=-4 lgE=-36
AsnGTT-001-N-3p-60-77 NT=37/37 RC=144/165 UH=74/89 lgP=-6 lgE=-35
AsnGTT-003-N-3p-60-77 NT=63/66 RC=138/158 UH=71/84 lgP=-7 lgE=-33
AsnGTT-013-NM-3p-57-76 NT=27/27 RC=56/62 UH=30/34 lgP=-6 lgE=-6
AsnGTT-013-NM-Mi-37-56 NT=31/31 RC=41/46 UH=31/36 lgP=-5 lgE=-3
AspGTC-001-N-3i-35-74 NT=59/64 RC=106/123 UH=61/72 lgP=-4 lgE=-31
AspGTC-001-N-3p-56-75 NT=37/37 RC=60/64 UH=39/42 lgP=-5 lgE=-15
AspGTC-001-N-Mi-10-36 NT=48/77 RC=116/185 UH=57/101 lgP=-5 lgE=-26
AspGTC-002-N-3i-35-74 NT=515/548 RC=1111/1462 UH=596/750 lgP=-4 lgE=∞
AspGTC-002-N-3p-58-75 NT=1065/1206 RC=4611/6008 UH=2051/2922 lgP=-6 lgE=∞
AspGTC-002-N-5i-1-36 NT=311/337 RC=766/885 UH=356/430 lgP=-5 lgE=-207
AspGTC-002-N-Mi-10-36 NT=417/1329 RC=1301/4788 UH=574/2367 lgP=-5 lgE=-216
CysGCA-001-N-3p-58-75 NT=652/668 RC=1689/6419 UH=801/1819 lgP=-7 lgE=∞

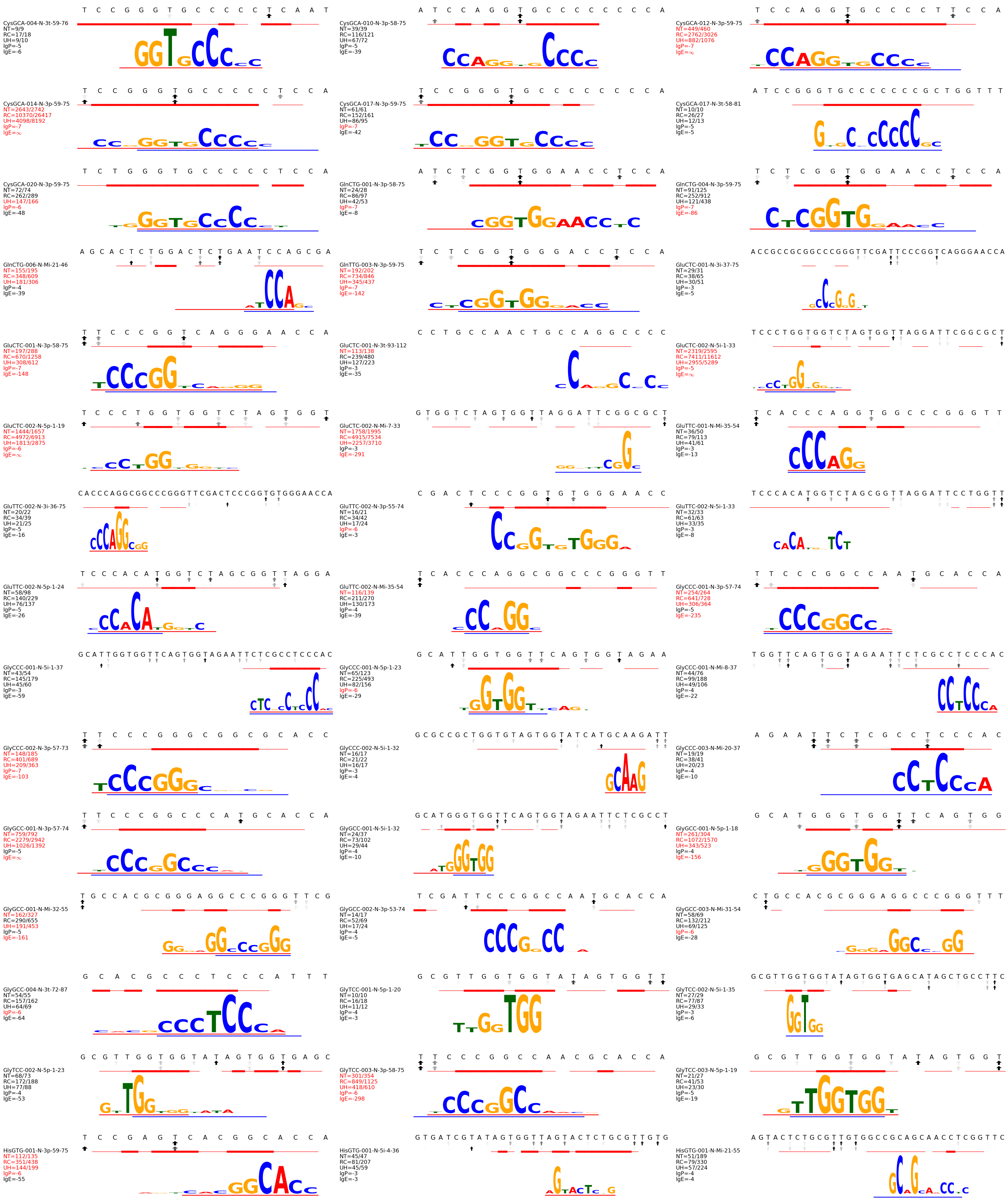

CysGCA-004-N-3t-59-76 NT=9/9 RC=17/18 UH=9/10 IgP=-5 IgE=-6
CysGCA-010-N-3p-58-75 NT=39/39 RC=116/121 UH=67/72 IgP=-5 IgE=-39
CysGCA-012-N-3p-59-75 NT=449/460 RC=2762/3026 UH=882/1076 IgP=-7 IgE=-∞
CysGCA-014-N-3p-59-75 NT=2643/2742 RC=10370/26417 UH=4098/8192 IgP=-7 IgE=-∞
CysGCA-017-N-3p-59-75 NT=61/61 RC=152/161 UH=86/95 IgP=-7 IgE=-42
CysGCA-017-N-3t-58-81 NT=10/10 RC=26/27 UH=12/13 IgP=-5 IgE=-5
CysGCA-020-N-3p-59-75 NT=72/74 RC=262/289 UH=147/166 IgP=-6 IgE=-48
GlnCTG-001-N-3p-58-75 NT=24/28 RC=86/97 UH=42/53 IgP=-7 IgE=-8
GlnCTG-004-N-3p-59-75 NT=91/125 RC=252/912 UH=121/438 IgP=-7 IgE=-86
GlnCTG-006-N-Mi-21-46 NT=155/195 RC=348/609 UH=181/306 IgP=-4 IgE=-39
GlnTTG-003-N-3p-59-75 NT=192/202 RC=734/846 UH=345/437 IgP=-7 IgE=-142
GluCTC-001-N-3i-37-75 NT=29/31 RC=38/65 UH=30/51 IgP=-3 IgE=-5
GluCTC-001-N-3p-58-75 NT=197/288 RC=670/1258 UH=308/612 IgP=-7 IgE=-148
GluCTC-001-N-3t-93-112 NT=113/138 RC=239/480 UH=127/223 IgP=-3 IgE=-35
GluCTC-002-N-5i-1-33 NT=2319/2595 RC=7411/11612 UH=2955/5289 IgP=-5 IgE=-∞
GluCTC-002-N-5p-1-19 NT=1444/1657 RC=4972/6913 UH=1813/2875 IgP=-6 IgE=-∞
GluCTC-002-N-Mi-7-33 NT=1758/1995 RC=4915/7534 UH=2257/3710 IgP=-3 IgE=-291
GluTTC-001-N-Mi-35-54 NT=36/50 RC=79/113 UH=41/61 IgP=-3 IgE=-13
GluTTC-002-N-3i-36-75 NT=20/22 RC=34/39 UH=21/25 IgP=-5 IgE=-16
GluTTC-002-N-3p-55-74 NT=16/21 RC=34/42 UH=17/24 IgP=-6 IgE=-3
GluTTC-002-N-5i-1-33 NT=32/33 RC=61/63 UH=33/35 IgP=-3 IgE=-8
GluTTC-002-N-5p-1-24 NT=58/98 RC=140/229 UH=76/137 IgP=-5 IgE=-26
GluTTC-002-N-Mi-35-54 NT=116/139 RC=211/270 UH=130/173 IgP=-4 IgE=-39
GlyCCC-001-N-3p-57-74 NT=254/264 RC=641/728 UH=306/364 IgP=-5 IgE=-235
GlyCCC-001-N-5i-1-37 NT=43/54 RC=145/179 UH=45/60 IgP=-3 IgE=-59
GlyCCC-001-N-5p-1-23 NT=65/123 RC=225/493 UH=82/156 IgP=-6 IgE=-29
GlyCCC-001-N-Mi-8-37 NT=44/76 RC=99/188 UH=49/106 IgP=-4 IgE=-22
GlyCCC-002-N-3p-57-73 NT=148/185 RC=401/689 UH=209/363 IgP=-7 IgE=-103
GlyCCC-002-N-5i-1-32 NT=16/17 RC=21/22 UH=16/17 IgP=-3 IgE=-4
GlyCCC-003-N-Mi-20-37 NT=19/19 RC=38/41 UH=20/23 IgP=-4 IgE=-10
GlyGCC-001-N-3p-57-74 NT=759/792 RC=2279/2942 UH=1026/1392 IgP=-5 IgE=-∞
GlyGCC-001-N-5i-1-32 NT=24/37 RC=73/102 UH=29/44 IgP=-4 IgE=-10
GlyGCC-001-N-5p-1-18 NT=261/304 RC=1072/1570 UH=343/523 IgP=-4 IgE=-156
GlyGCC-001-N-Mi-32-55 NT=162/327 RC=290/655 UH=191/453 IgP=-5 IgE=-161
GlyGCC-002-N-3p-53-74 NT=14/17 RC=52/69 UH=17/24 IgP=-4 IgE=-5
GlyGCC-003-N-Mi-31-54 NT=58/69 RC=132/212 UH=69/125 IgP=-6 IgE=-28
GlyGCC-004-N-3t-72-87 NT=54/55 RC=157/162 UH=64/69 IgP=-6 IgE=-64
GlyTCC-001-N-5p-1-20 NT=10/10 RC=16/18 UH=11/12 IgP=-4 IgE=-3
GlyTCC-002-N-5i-1-35 NT=27/29 RC=77/87 UH=29/33 IgP=-3 IgE=-6
GlyTCC-002-N-5p-1-23 NT=68/73 RC=172/188 UH=77/88 IgP=-4 IgE=-53
GlyTCC-003-N-3p-58-75 NT=301/354 RC=849/1125 UH=418/610 IgP=-6 IgE=-298
GlyTCC-003-N-5p-1-19 NT=21/27 RC=41/53 UH=23/30 IgP=-5 IgE=-19
HisGTG-001-N-3p-59-75 NT=112/135 RC=351/438 UH=144/199 IgP=-6 IgE=-55
HisGTG-001-N-5i-4-36 NT=45/47 RC=81/207 UH=45/59 IgP=-3 IgE=-3
HisGTG-001-N-Mi-21-55 NT=51/189 RC=79/330 UH=57/224 IgP=-4 IgE=-4

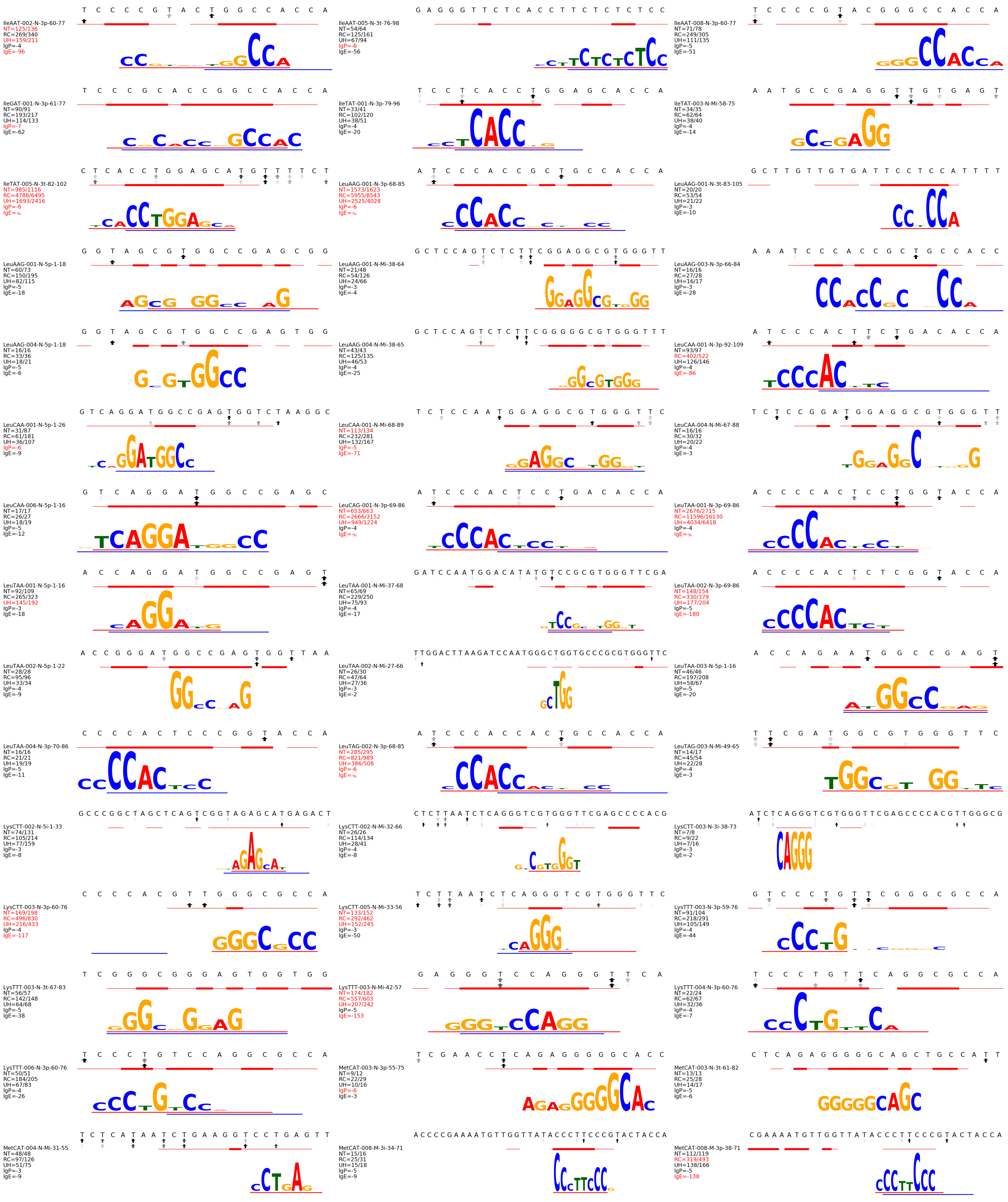
T C C C C G T A C T G G C C A C C A
IleAAT-002-N-3p-60-77 NT=125/136 RC=269/340 UH=159/211 IgP=-4 IgE=-96
G A G G G T T C T C A C C T T C T C T C T C C
IleAAT-005-N-3t-76-98 NT=54/64 RC=125/161 UH=67/94 IgP=-6 IgE=-56
T C C C C G T A C G G G C C A C C A
IleAAT-008-N-3p-60-77 NT=71/78 RC=249/305 UH=111/135 IgP=-5 IgE=-51
T C C C C G C A C C G G C C A C C A
IleGAT-001-N-3p-61-77 NT=90/91 RC=193/217 UH=114/133 IgP=-7 IgE=-62
T C C T C A C C T G G A G C A C C A
IleTAT-001-N-3p-79-96 NT=33/41 RC=102/120 UH=38/51 IgP=-4 IgE=-20
A A T G C C G A G G T T G T G A G T
IleTAT-003-N-Mi-58-75 NT=34/35 RC=62/64 UH=38/40 IgP=-4 IgE=-14
C T C A C C T G G A G C A T G T T T T C T
IleTAT-005-N-3t-82-102 NT=985/1116 RC=4788/6495 UH=1693/2416 IgP=-6 IgE=-∞
A T C C C A C C G C T G C C A C C A
LeuAAG-001-N-3p-68-85 NT=1573/1623 RC=5955/8543 UH=2525/4028 IgP=-6 IgE=-∞
G C T T G T T G T G A T T C C T C C A T T T T
LeuAAG-001-N-3t-83-105 NT=20/20 RC=53/54 UH=21/22 IgP=-3 IgE=-10
G G T A G C G T G G C C G A G C G G
LeuAAG-001-N-5p-1-18 NT=60/73 RC=150/195 UH=82/115 IgP=-5 IgE=-18
G C T C C A G T C T C T T C G G A G G C G T G G G T T
LeuAAG-001-N-Mi-38-64 NT=21/48 RC=54/126 UH=24/66 IgP=-3 IgE=-4
A A A T C C C A C C G C T G C C A C C
LeuAAG-003-N-3p-66-84 NT=16/16 RC=27/28 UH=16/17 IgP=-3 IgE=-28
G G T A G C G T G G C C G A G T G G
LeuAAG-004-N-5p-1-18 NT=16/16 RC=33/36 UH=18/21 IgP=-5 IgE=-6
G C T C C A G T C T C T T C G G G G G C G T G G G T T
LeuAAG-004-N-Mi-38-65 NT=43/43 RC=125/135 UH=46/53 IgP=-4 IgE=-25
A T C C C A C T T C T G A C A C C A
LeuCAA-001-N-3p-92-109 NT=93/97 RC=402/522 UH=126/146 IgP=-4 IgE=-86
G T C A G G A T G G C C G A G T G G T C T A A G G C
LeuCAA-001-N-5p-1-26 NT=31/87 RC=61/181 UH=36/107 IgP=-6 IgE=-9
T C T C C A A T G G A G G C G T G G G T T C
LeuCAA-001-N-Mi-68-89 NT=113/134 RC=232/281 UH=132/167 IgP=-5 IgE=-71
T C T C C G G A T G G A G G C G T G G G T T
LeuCAA-004-N-Mi-67-88 NT=16/16 RC=30/32 UH=20/22 IgP=-4 IgE=-3
G T C A G G A T G G C C G A G C
LeuCAA-006-N-5p-1-16 NT=17/17 RC=26/27 UH=18/19 IgP=-5 IgE=-12
A T C C C A C T C C T G A C A C C A
LeuCAG-001-N-3p-69-86 NT=653/663 RC=2666/3152 UH=949/1224 IgP=-4 IgE=-∞
A C C C C A C T C C T G G T A C C A
LeuTAA-001-N-3p-69-86 NT=2676/2715 RC=11596/16130 UH=4034/6418 IgP=-4 IgE=-∞
A C C A G G A T G G C C G A G T
LeuTAA-001-N-5p-1-16 NT=92/109 RC=265/323 UH=145/192 IgP=-3 IgE=-18
G A T C C A A T G G A C A T A T G T C C G C G T G G G T T C G A
LeuTAA-001-N-Mi-37-68 NT=65/69 RC=229/250 UH=75/93 IgP=-4 IgE=-17
A C C C C A C T C T C G G T A C C A
LeuTAA-002-N-3p-69-86 NT=148/154 RC=330/379 UH=177/204 IgP=-5 IgE=-180
A C C G G G A T G G C C G A G T G G T T A A
LeuTAA-002-N-5p-1-22 NT=28/28 RC=95/96 UH=33/34 IgP=-4 IgE=-9
TTGGACTTAAGATCCAATGGGCTGGTGCCCGCGTGGGTTC
LeuTAA-002-N-Mi-27-66 NT=26/30 RC=47/64 UH=27/36 IgP=-3 IgE=-2
A C C A G A A T G G C C G A G T
LeuTAA-003-N-5p-1-16 NT=46/46 RC=197/208 UH=58/67 IgP=-5 IgE=-20
C C C C A C T C C C G G T A C C A
LeuTAA-004-N-3p-70-86 NT=16/16 RC=21/21 UH=19/19 IgP=-5 IgE=-11
A T C C C A C C A C T G C C A C C A
LeuTAG-002-N-3p-68-85 NT=285/295 RC=821/989 UH=386/508 IgP=-6 IgE=-∞
T T C G A T G G C G T G G G T T C
LeuTAG-003-N-Mi-49-65 NT=14/17 RC=45/54 UH=22/28 IgP=-4 IgE=-3
GCCCGGCTAGCTCAGTCGGTAGAGCATGAGACT
LysCTT-002-N-5i-1-33 NT=74/131 RC=105/214 UH=77/159 IgP=-3 IgE=-8
CTCTTAATCTCAGGGTCGTGGGTTCGAGCCCCACG
LysCTT-002-N-Mi-32-66 NT=26/26 RC=114/134 UH=28/41 IgP=-4 IgE=-8
ATCTCAGGGTCGTGGGTTCGAGCCCCACGTTGGGCG
LysCTT-003-N-3i-38-73 NT=7/8 RC=9/22 UH=7/16 IgP=-3 IgE=-2
C C C C A C G T T G G G C G C C A
LysCTT-003-N-3p-60-76 NT=169/198 RC=496/830 UH=216/433 IgP=-4 IgE=-117
T C T T A A T C T C A G G G T C G T G G G T T C
LysCTT-005-N-Mi-33-56 NT=133/152 RC=292/462 UH=152/245 IgP=-3 IgE=-50
G T C C C T G T T C G G G C G C C A
LysTTT-003-N-3p-59-76 NT=91/104 RC=218/291 UH=105/149 IgP=-4 IgE=-44
T C G G G C G G G A G T G G T G G
LysTTT-003-N-3t-67-83 NT=56/57 RC=142/148 UH=64/68 IgP=-5 IgE=-38
G A G G G T C C A G G G T T C A
LysTTT-003-N-Mi-42-57 NT=174/182 RC=557/603 UH=207/242 IgP=-5 IgE=-153
T C C C T G T T C A G G C G C A
LysTTT-004-N-3p-60-76 NT=22/24 RC=62/67 UH=32/36 IgP=-4 IgE=-7
T C C C T G T C C A G G C G C C A
LysTTT-006-N-3p-60-76 NT=50/51 RC=184/205 UH=67/83 IgP=-4 IgE=-26
T C G A A C C T C A G A G G G G G C A C C
MetCAT-003-N-3p-55-75 NT=9/12 RC=22/29 UH=10/16 IgP=-6 IgE=-3
C T C A G A G G G G G C A G C T G C C A T T
MetCAT-003-N-3t-61-82 NT=13/13 RC=25/28 UH=14/17 IgP=-5 IgE=-6
T C T C A T A A T C T G A A G G T C C T G A G T T
MetCAT-004-N-Mi-31-55 NT=48/48 RC=97/126 UH=51/75 IgP=-3 IgE=-9
ACCCCGAAAATGTTGGTTATACCCTTCCCCGTACTACCA
MetCAT-008-M-3i-34-71 NT=15/16 RC=25/31 UH=15/18 IgP=-5 IgE=-9
CGAAAATGTTGGTTATACCCTTCCCGTACTACCA
MetCAT-008-M-3p-38-71 NT=112/119 RC=319/493 UH=138/166 IgP=-5 IgE=-138

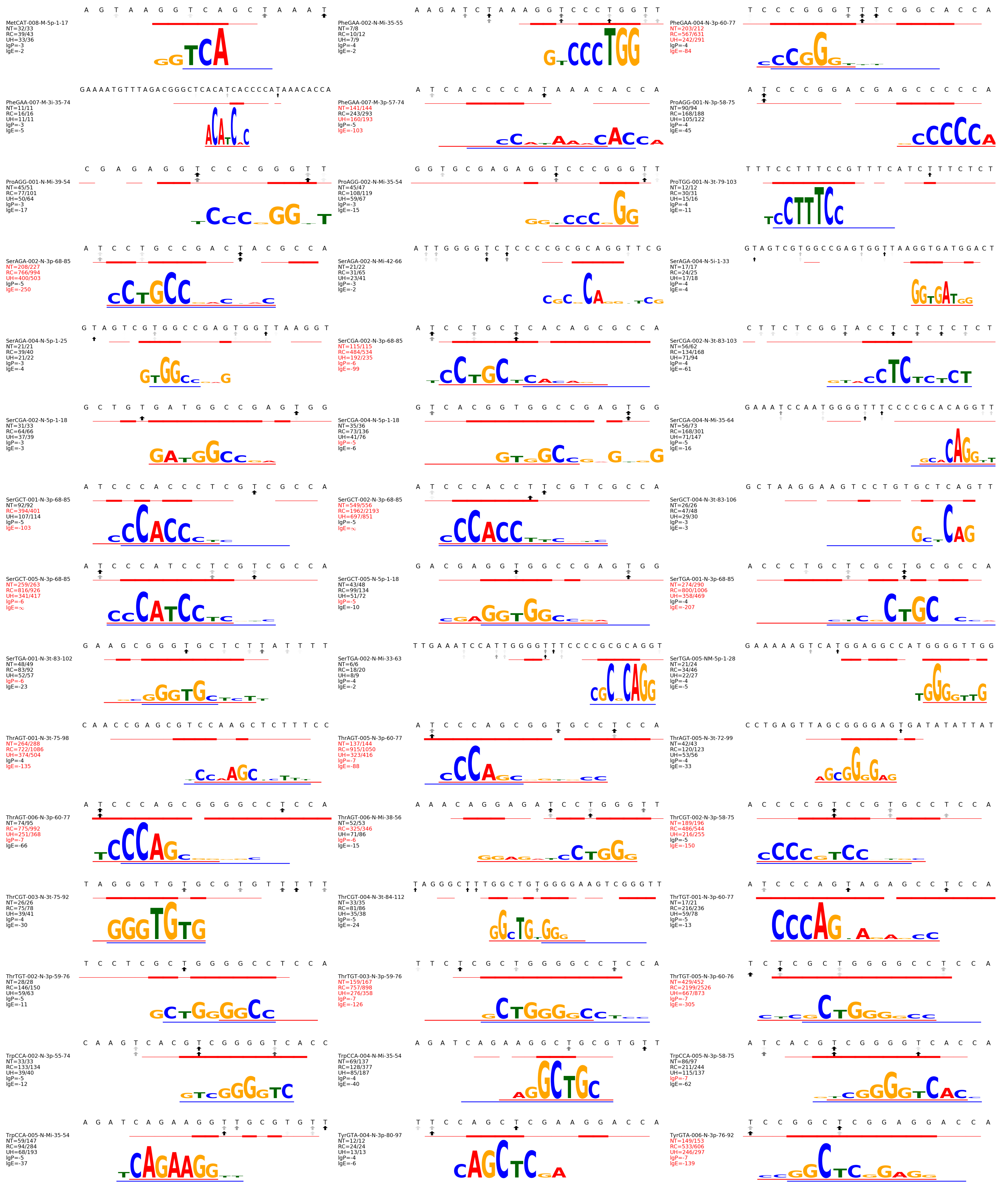
AGTAAGGTCAGCTAAAT
MetCAT-008-M-5p-1-17
NT=32/33
RC=39/43
UH=33/36
lgP=-3
lgE=-2
AAGATCTAAAGGTCCCTGGTT
PheGAA-002-N-Mi-35-55
NT=7/8
RC=10/12
UH=7/9
lgP=-4
lgE=-2
TCCCGGGTTTCGGCACCA
PheGAA-004-N-3p-60-77
NT=203/212
RC=567/631
UH=242/291
lgP=-4
lgE=-84
GAAAATGTTTAGACGGGCTCACATCACCCCATAAACACCA
PheGAA-007-M-3i-35-74
NT=11/11
RC=16/16
UH=11/11
lgP=-3
lgE=-5
ATCACCCCATAAACACCA
PheGAA-007-M-3p-57-74
NT=141/144
RC=243/293
UH=160/193
lgP=-5
lgE=-103
ATCCCGGACGAGCCCCCA
ProAGG-001-N-3p-58-75
NT=90/94
RC=168/188
UH=105/122
lgP=-4
lgE=-45
CGAGAGGTCCCGGGTT
ProAGG-001-N-Mi-39-54
NT=45/51
RC=77/101
UH=50/64
lgP=-3
lgE=-17
GGTGCGAGAGGTCCCGGGTT
ProAGG-002-N-Mi-35-54
NT=45/47
RC=108/119
UH=59/67
lgP=-3
lgE=-15
TTTCCTTTCCGTTTCATCTTTTCTCT
ProTGG-001-N-3t-79-103
NT=12/12
RC=30/31
UH=15/16
lgP=-4
lgE=-11
ATCCTGCCGACTACGCCA
SerAGA-002-N-3p-68-85
NT=208/227
RC=766/994
UH=400/503
lgP=-5
lgE=-250
ATTGGGGTCTCCCCGCGCAGGTTCG
SerAGA-002-N-Mi-42-66
NT=21/22
RC=31/65
UH=23/41
lgP=-3
lgE=-2
GTAGTCGTGGCCGAGTGGTTAAGGTGATGGACT
SerAGA-004-N-5i-1-33
NT=17/17
RC=24/25
UH=17/18
lgP=-4
lgE=-4
GTAGTCGTGGCCGAGTGGTTAAGGT
SerAGA-004-N-5p-1-25
NT=21/21
RC=39/40
UH=21/22
lgP=-3
lgE=-4
ATCCTGCTCACAGCGCCA
SerCGA-002-N-3p-68-85
NT=115/115
RC=484/534
UH=192/235
lgP=-6
lgE=-99
CTTCTCGGTACCTCTCTCT
SerCGA-002-N-3t-83-103
NT=56/62
RC=134/168
UH=71/94
lgP=-4
lgE=-61
GCTGTGATGGCCGAGTGG
SerCGA-002-N-5p-1-18
NT=31/33
RC=64/66
UH=37/39
lgP=-3
lgE=-3
GTCACGGTGGCCGAGTGG
SerCGA-004-N-5p-1-18
NT=35/36
RC=73/136
UH=41/76
lgP=-5
lgE=-6
GAAATCCAATGGGGTTTCCCCGCACAGGTT
SerCGA-004-N-Mi-35-64
NT=56/73
RC=168/301
UH=71/147
lgP=-5
lgE=-16
ATCCCACCCTCGTCGCCA
SerGCT-001-N-3p-68-85
NT=92/92
RC=394/401
UH=107/114
lgP=-5
lgE=-103
ATCCCACCTTCGTCGCCA
SerGCT-002-N-3p-68-85
NT=549/556
RC=1962/2193
UH=697/851
lgP=-5
lgE=∞
GCTAAGGAAGTCCTGTGCTCAGTT
SerGCT-004-N-3t-83-106
NT=26/26
RC=47/48
UH=29/30
lgP=-3
lgE=-3
ATCCCATCCTCGTCGCCA
SerGCT-005-N-3p-68-85
NT=259/263
RC=816/926
UH=341/417
lgP=-6
lgE=∞
GACGAGGTGGCCGAGTGG
SerGCT-005-N-5p-1-18
NT=43/48
RC=99/134
UH=51/72
lgP=-5
lgE=-10
ACCCTGCTCGCTGCGCCA
SerTGA-001-N-3p-68-85
NT=274/290
RC=800/1006
UH=358/469
lgP=-4
lgE=-207
GAAGCGGGTGCTCTTATTTT
SerTGA-001-N-3t-83-102
NT=48/49
RC=83/92
UH=52/57
lgP=-6
lgE=-23
TTGAAATCCATTGGGGTTTCCCCGCGCAGGT
SerTGA-002-N-Mi-33-63
NT=6/6
RC=18/20
UH=8/9
lgP=-4
lgE=-2
GAAAAGTCATGGAGGCCATGGGGTTGG
SerTGA-005-NM-5p-1-28
NT=21/24
RC=34/46
UH=22/27
lgP=-4
lgE=-5
CAACCGAGCGTCCAAGCTCTTTCC
ThrAGT-001-N-3t-75-98
NT=264/288
RC=722/1086
UH=374/504
lgP=-4
lgE=-135
ATCCCAGCGGTGCCTCCA
ThrAGT-005-N-3p-60-77
NT=137/144
RC=915/1050
UH=323/416
lgP=-7
lgE=-88
CCTGAGTTAGCGGGGAGTGATATATTAT
ThrAGT-005-N-3t-72-99
NT=42/43
RC=120/123
UH=53/56
lgP=-4
lgE=-33
ATCCCAGCGGGGCCTCCA
ThrAGT-006-N-3p-60-77
NT=74/95
RC=775/992
UH=251/368
lgP=-7
lgE=-66
AAACAGGAGATCCTGGGTT
ThrAGT-006-N-Mi-38-56
NT=52/53
RC=325/346
UH=71/86
lgP=-6
lgE=-15
ACCCCGTCCGTGCCTCCA
ThrCGT-002-N-3p-58-75
NT=189/196
RC=486/544
UH=216/255
lgP=-5
lgE=-150
TAGGGTGTGCGTGTTTTT
ThrCGT-003-N-3t-75-92
NT=26/26
RC=75/78
UH=39/41
lgP=-4
lgE=-30
TAGGGCTTTGGCTGTGGGGAAGTCGGGTT
ThrCGT-004-N-3t-84-112
NT=33/35
RC=81/86
UH=35/38
lgP=-5
lgE=-24
ATCCCAGTAGAGCCTCCA
ThrTGT-001-N-3p-60-77
NT=17/21
RC=216/236
UH=59/78
lgP=-5
lgE=-13
TCCTCGCTGGGGCCTCCA
ThrTGT-002-N-3p-59-76
NT=28/28
RC=146/150
UH=59/63
lgP=-5
lgE=-11
TTCTCGCTGGGGCCTCCA
ThrTGT-003-N-3p-59-76
NT=159/167
RC=757/898
UH=276/358
lgP=-7
lgE=-126
TCTCGCTGGGGCCTCCA
ThrTGT-005-N-3p-60-76
NT=429/452
RC=2199/2526
UH=667/873
lgP=-7
lgE=-305
CAAGTCACGTCGGGGTCACC
TrpCCA-002-N-3p-55-74
NT=33/33
RC=133/134
UH=39/40
lgP=-5
lgE=-12
AGATCAGAAGGCTGCGTGTT
TrpCCA-004-N-Mi-35-54
NT=69/137
RC=128/377
UH=85/187
lgP=-4
lgE=-40
ATCACGTCGGGGTCACCA
TrpCCA-005-N-3p-58-75
NT=86/97
RC=211/244
UH=115/137
lgP=-7
lgE=-62
AGATCAGAAGGTTGCGTGTT
TrpCCA-005-N-Mi-35-54
NT=59/147
RC=94/284
UH=68/193
lgP=-5
lgE=-37
TTCCAGCTCGAAGGACCA
TyrGTA-004-N-3p-80-97
NT=12/12
RC=24/24
UH=13/13
lgP=-4
lgE=-6
TCCGGCTCGGAGGACCA
TyrGTA-006-N-3p-76-92
NT=149/153
RC=533/606
UH=246/297
lgP=-7
lgE=-139

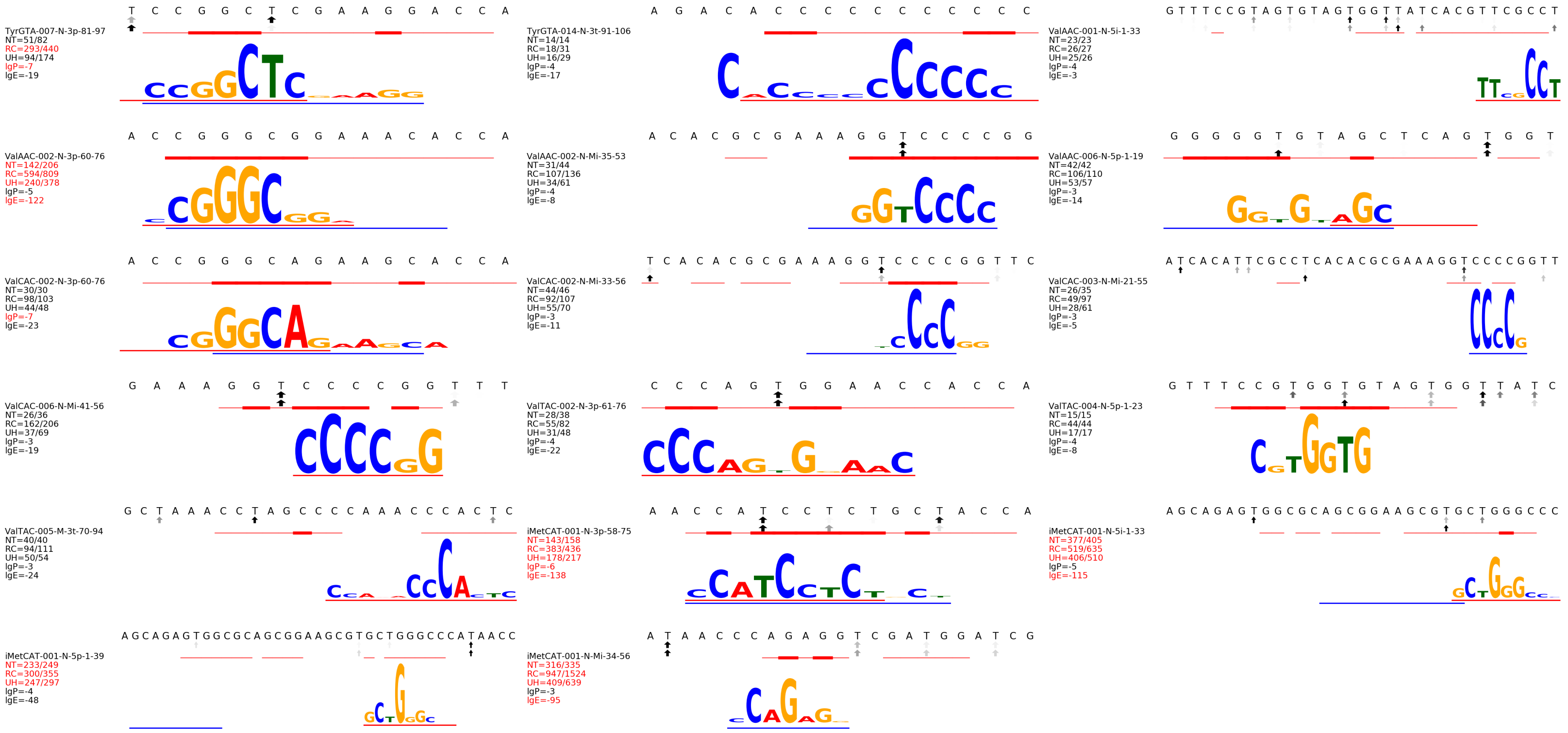

T C C G G C T C G A A G G A C C A
TyrGTA-007-N-3p-81-97
NT=51/82
RC=293/440
UH=94/174
lgP=-7
lgE=-19
A G A C A C C C C C C C C C C C
TyrGTA-014-N-3t-91-106
NT=14/14
RC=18/31
UH=16/29
lgP=-4
lgE=-17
G T T T C C G T A G T G T A G T G G T T A T C A C G T T C G C C T
ValAAC-001-N-5i-1-33
NT=23/23
RC=26/27
UH=25/26
lgP=-4
lgE=-3
A C C G G G C G G A A A C A C C A
ValAAC-002-N-3p-60-76
NT=142/206
RC=594/809
UH=240/378
lgP=-5
lgE=-122
A C A C G C G A A A G G T C C C C G G
ValAAC-002-N-Mi-35-53
NT=31/44
RC=107/136
UH=34/61
lgP=-4
lgE=-8
G G G G G T G T A G C T C A G T G G T
ValAAC-006-N-5p-1-19
NT=42/42
RC=106/110
UH=53/57
lgP=-3
lgE=-14
A C C G G G C A G A A G C A C C A
ValCAC-002-N-3p-60-76
NT=30/30
RC=98/103
UH=44/48
lgP=-7
lgE=-23
T C A C A C G C G A A A G G T C C C C G G T T C
ValCAC-002-N-Mi-33-56
NT=44/46
RC=92/107
UH=55/70
lgP=-3
lgE=-11
A T C A C A T T C G C C T C A C A C G C G A A A G G T C C C C G G T T
ValCAC-003-N-Mi-21-55
NT=26/35
RC=49/97
UH=28/61
lgP=-3
lgE=-5
G A A A G G T C C C C G G T T T
ValCAC-006-N-Mi-41-56
NT=26/36
RC=162/206
UH=37/69
lgP=-3
lgE=-19
C C C A G T G G A A C C A C C A
ValTAC-002-N-3p-61-76
NT=28/38
RC=55/82
UH=31/48
lgP=-4
lgE=-22
G T T T C C G T G G T G T A G T G G T T A T C
ValTAC-004-N-5p-1-23
NT=15/15
RC=44/44
UH=17/17
lgP=-4
lgE=-8
G C T A A A C C T A G C C C C A A A C C C A C T C
ValTAC-005-M-3t-70-94
NT=40/40
RC=94/111
UH=50/54
lgP=-3
lgE=-24
A A C C A T C C T C T G C T A C C A
iMetCAT-001-N-3p-58-75
NT=143/158
RC=383/436
UH=178/217
lgP=-6
lgE=-138
A G C A G A G T G G C G C A G C G G A A G C G T G C T G G G C C C
iMetCAT-001-N-5i-1-33
NT=377/405
RC=519/635
UH=406/510
lgP=-5
lgE=-115
A G C A G A G T G G C G C A G C G G A A G C G T G C T G G G C C C A T A A C C
iMetCAT-001-N-5p-1-39
NT=233/249
RC=300/355
UH=247/297
lgP=-4
lgE=-48
A T A A C C C A G A G G T C G A T G G A T C G
iMetCAT-001-N-Mi-34-56
NT=316/335
RC=947/1524
UH=409/639
lgP=-3
lgE=-95
