# Supplementary material for: Large-Scale Computational Discovery of Binding Motifs in tRNA Fragments
Source: Front Mol Biosci. 2021 Jun 22;8:647449. doi: 10.3389/fmolb.2021.647449 (PMC8258673; doi:10.3389/fmolb.2021.647449)
Supplement: Supplementary file 2 [file DataSheet1.DOCX]

Supplementary Material

# Supplementary Figures and Tables

## Supplementary Figures


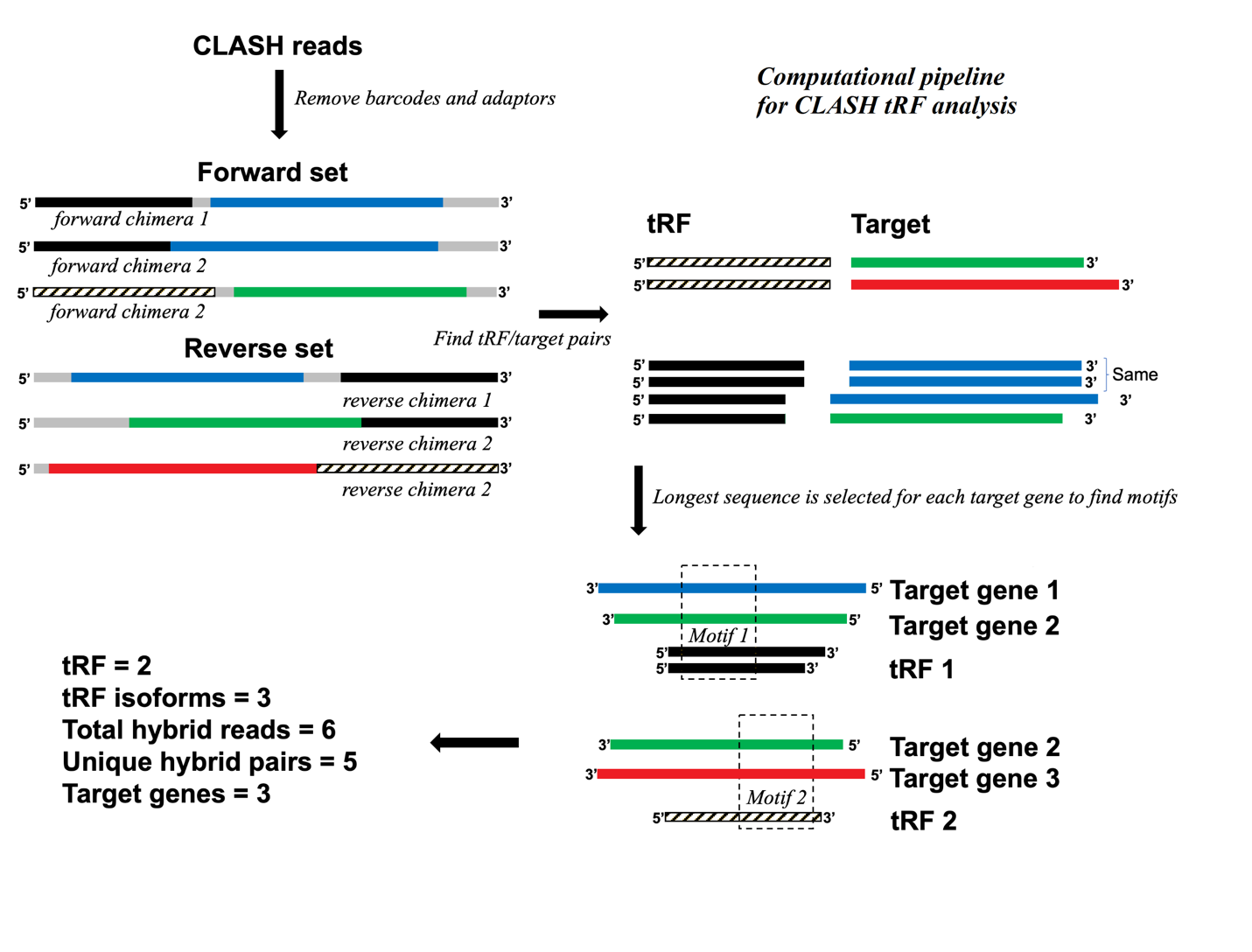


**Supplementary Figure 1.** Computational analysis pipeline. Solid black segments indicate isoforms of a specific tRF type (such as 5p) aligned to the same tRNA sequence. These isoforms of different lengths are categorized as a single tRF. Striped, black segments indicate isoforms from another tRNA sequence. Blue, green, and red segments indicate target sequences mapped to three different genes. Only the longest sequences of every target gene are combined to find motif for a given tRF. A proposed model for the chimera formation is illustrated in Fig. 3D.


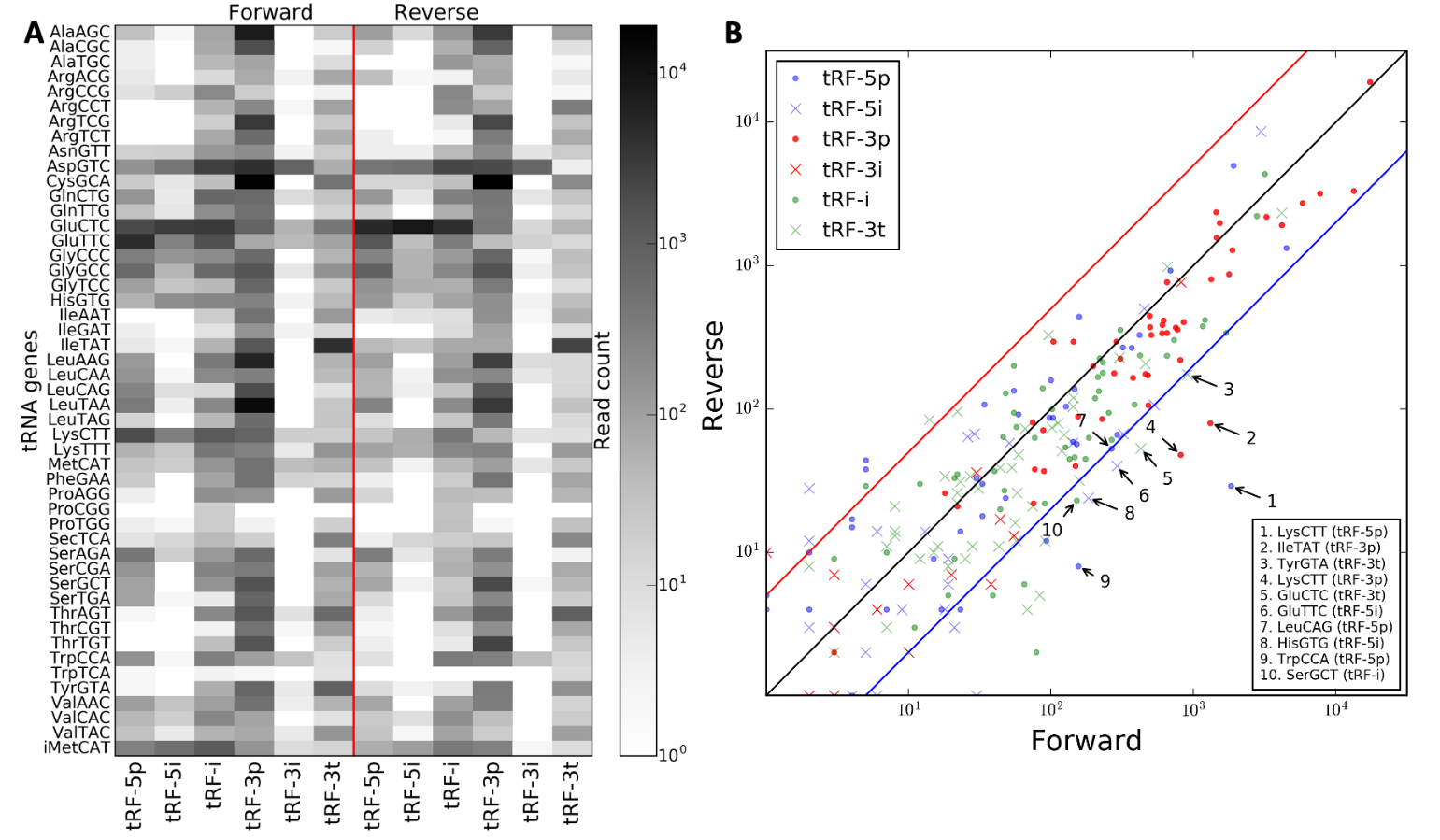


**Supplementary Figure 2.** Anticodon abundance across varying mapped regions in forward set and reverse set. (A) A heatmap of the total abundance of tRFs formed from different tRNA isoacceptors. (B) The same data presented as a scatter plot for tRNA isoacceptors in forward versus reverse pairs. The 10 most abundant tRFs with the highest differential frequencies in the two orientations (fold-change > 5) are labeled.


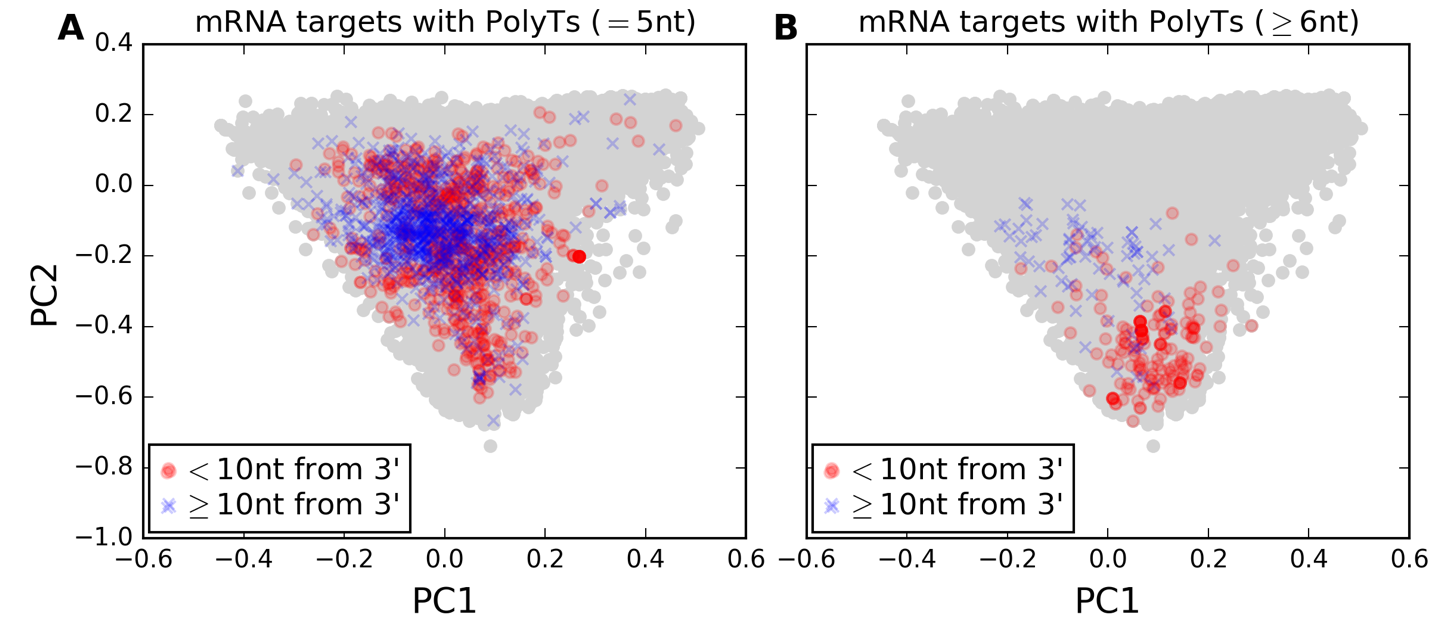


**Supplementary Figure 3.** PCA of dinucleotide frequencies in selected mRNA targets with **(A)** 5-nt continuous poly-T runs and **(B)** >5-nt continuous poly-T runs. The grey shape shows the full PCA plot (see Fig. 2C).


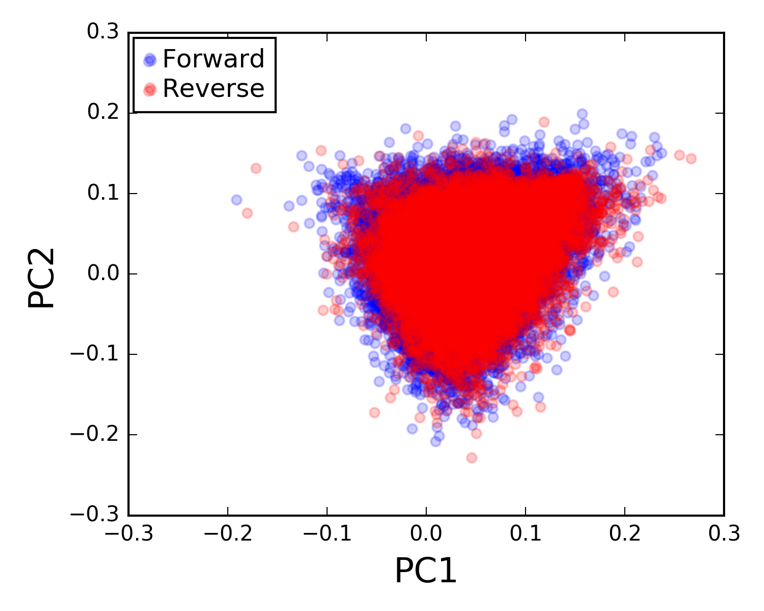


**Supplementary Figure 4.** PCA plots of dinucleotide frequencies in tRF-mRNA pairs with motif interactions.

**Supplementary Figure 5.** Hybridization patterns in tRFs (***see PDF file, 5 pages***). The bitscore of the nucleotide matched to the tRF sequence in the motifs detected in forward and reverse pairs is shown in the logo. The red line shows hybridizing tRF-target nucleotides (thick lines if present in >80%, and thin lines in >50% of the targets). Red and blue lines underline the motifs identified using only forward or reverse pairs, respectively. Two lines of arrows show the T→C conversion sites identified in human (top) and mouse (bottom) PAR-CLIP datasets with frequencies depicted as varying from black (highest RPM) to gray (lower RPM). NT: number of unique target genes. RC: total number of CLASH reads. UH: unique tRF-target hybrids in CLASH. Numbers before slash indicate tRF-mRNA pairs having motif interactions and numbers after slash indicate all pairs. lgP and lgE: base 10 logarithms of e-value (MEME) and p-value (FIMO) of motif. The top 1/3 values in each category are shown in red lettering.

## Supplementary Tables

**Supplementary Table 1.** Number of reads supporting tRF-tRF hybrids.

|  | tRF-5p (3') | tRF-5i (3') | tRF-3p (3') | tRF-3i (3') | tRF-3t (3') | tRF-i (3') |
| --- | --- | --- | --- | --- | --- | --- |
| tRF-5p (5') | 200 (163) | 258 (247) | 5,804 (4,984) | 41 (25) | 887 (635) | 53,806 (51,611) |
| tRF-5i (5') | 18 (12) | 1 (0) | 1,588 (1,413) | 10 (4) | 129 (75) | 877 (737) |
| tRF-3p (5') | 11,015 (10,361) | 6,845 (6,683) | 2,434 (1,967) | 1,792 (1,706) | 1,387 (987) | 11,274 (9,753) |
| tRF-3i (5') | 75 (51) | 37 (31) | 758 (687) | 57 (56) | 263 (168) | 158 (111) |
| tRF-3t (5') | 1,482 (1,342) | 437 (395) | 278 (192) | 24 (16) | 32,784 (32,447) | 677 (485) |
| tRF-i (5') | 3,620 (3,161) | 2,558 (2,430) | 415,548 (408,520) | 657 (563) | 25,205 (21,206) | 31,778 (28,079) |

Rows designate tRFs on the 5’ end of chimeras, columns – 3’ end. Numbers in parentheses indicate the number of hybrids supported by at least two reads.

**Supplementary Table 2.** Top 20 tRF and mRNA interactions in forward pairs and reverse pairs

|  | tRF | Sequence | Target Gene | Read Count | Fraction of pairs (%) | Cumulative fraction (%) |
| --- | --- | --- | --- | --- | --- | --- |
| Forward pairs | CysGCA-014-N-3p | TCCGGGTGCCCCCTCCA | HIST2H2AA3 | 2422 | 2.01 | 2.01 |
|  | IleTAT-003-N-3p | AGCCTCACCTGGAGCA | CPD | 1028 | 0.85 | 2.86 |
|  | ThrAGT-005-N-3p | ATCCCAGCGGTGCCTCCA | RPL35A | 482 | 0.4 | 3.26 |
|  | CysGCA-001-N-3p | TCCAGGTGCCCCCTCCA | HIST2H2AA3 | 449 | 0.37 | 3.64 |
|  | CysGCA-012-N-3p | TCCAGGTGCCCCTTCCA | HIST2H2AA3 | 411 | 0.34 | 3.98 |
|  | IleTAT-005-N-3t | CTCACCTGGAGCATGTTTTCT | RPL4 | 403 | 0.33 | 4.31 |
|  | ThrAGT-006-N-3p | ATCCCAGCGGGGCCTCCA | RPL35A | 396 | 0.33 | 4.64 |
|  | LeuAAG-001-N-3p | ATCCCACCGCTGCCACCA | DCTPP1 | 305 | 0.25 | 4.89 |
|  | GluTTC-003-N-5p | TCCCTGGTGGTCTAGTGGCTA | MAP2K7 | 295 | 0.24 | 5.14 |
|  | GluTTC-003-N-i | AGGATTCGGCGCTTTCACCGCCGCG | ERCC1 | 288 | 0.24 | 5.38 |
|  | AspGTC-002-N-3p | TTCCCCGACGGGGAGCCA | TIMM10 | 283 | 0.23 | 5.61 |
|  | LysCTT-003-N-i | AGGGTCGTGGGTTCGAGCCCCACGT | RBFOX1 | 252 | 0.21 | 5.82 |
|  | LeuTAA-001-N-3p | ACCCCACTCCTGGTACCA | ND6 | 248 | 0.21 | 6.03 |
|  | GlnCTG-004-N-3p | TCTCGGTGGAACCTCCA | HIST2H3A | 225 | 0.19 | 6.21 |
|  | SerAGA-002-N-3p | ATCCTGCCGACTACGCCA | HIST2H3A | 204 | 0.17 | 6.38 |
|  | CysGCA-014-N-3p | TCCGGGTGCCCCCTCCA | SYNGR2 | 192 | 0.16 | 6.54 |
|  | GluCTC-002-N-i | ACCGCCGCGGCCCGGGTT | ERCC1 | 181 | 0.15 | 6.69 |
|  | CysGCA-014-N-3p | TCCGGGTGCCCCCTCCA | ENO1 | 160 | 0.13 | 6.83 |
|  | GlyGCC-001-N-5p | GCATGGGTGGTTCAGTGGTA | EEF1A1 | 155 | 0.13 | 6.96 |
|  | ThrTGT-005-N-3p | TCTCGCTGGGGCCTCCA | HIST2H4A | 145 | 0.12 | 7.08 |
| Reverse pairs | CysGCA-014-N-3p | TCCGGGTGCCCCCTCCA | HIST2H2AA3 | 9645 | 11.15 | 11.15 |
|  | CysGCA-001-N-3p | ATCCAGGTGCCCCCTCC | HIST2H2AA3 | 3988 | 4.61 | 15.75 |
|  | CysGCA-012-N-3p | TCCAGGTGCCCCTTCCA | HIST2H2AA3 | 1273 | 1.47 | 17.23 |
|  | ThrTGT-005-N-3p | TCTCGCTGGGGCCTCC | HIST2H4A | 893 | 1.03 | 18.26 |
|  | ThrAGT-006-N-3p | ATCCCAGCGGGGCCTCCA | RPL35A | 390 | 0.45 | 18.71 |
|  | ThrTGT-003-N-3p | TTCTCGCTGGGGCCTCC | HIST2H4A | 346 | 0.4 | 19.11 |
|  | GlnCTG-004-N-3p | TCTCGGTGGAACCTCCA | HIST2H3A | 276 | 0.32 | 19.43 |
|  | GluCTC-002-N-5p | TCCCTGGTGGTCTAGTGGT | PKM | 251 | 0.29 | 19.72 |
|  | GlnTTG-003-N-3p | TCTCGGTGGGACCTCCA | HIST2H3A | 212 | 0.24 | 19.96 |
|  | AspGTC-002-N-5p | TCCTCGTTAGTATAGTGGTGA | TIMM23B | 193 | 0.22 | 20.18 |
|  | ThrAGT-005-N-3p | ATCCCAGCGGTGCCTCCA | RPL35A | 169 | 0.2 | 20.38 |
|  | LeuCAG-001-N-3p | ATCCCACTCCTGACACCA | ATP5G3 | 167 | 0.19 | 20.57 |
|  | ArgTCG-002-N-3p | ATCCCTCCGTGGTTACCA | RPS15A | 164 | 0.19 | 20.76 |
|  | LeuTAA-001-N-3p | ACCCCACTCCTGGTACCA | ATP5G3 | 156 | 0.18 | 20.94 |
|  | GluCTC-002-N-i | GTGGTCTAGTGGTTAGGATTCGGCGCT | SHTN1 | 153 | 0.18 | 21.12 |
|  | IleTAT-005-N-3t | CTCACCTGGAGCATGTTTTCT | PPP1R9A | 149 | 0.17 | 21.29 |
|  | GlyGCC-001-N-5p | GCATGGGTGGTTCAGTGG | PSMB3 | 146 | 0.17 | 21.46 |
|  | IleTAT-005-N-3t | CTCACCTGGAGCATGTTTTCT | TRAF5 | 137 | 0.16 | 21.62 |
|  | GluTTC-003-N-5p | TCCCTGGTGGTCTAGTGGC | RPSA | 127 | 0.15 | 21.77 |
|  | GlyGCC-001-N-5p | GCATGGGTGGTTCAGTGG | RPL4 | 127 | 0.15 | 21.91 |
